# Validation of ELISAs for Isoflavones and Enterolactone for Phytoestrogen Intake Assessment in the French Population

**DOI:** 10.3390/nu15040967

**Published:** 2023-02-15

**Authors:** Souad Bensaada, Isabelle Raymond, Isabelle Pellegrin, Jean-François Viallard, Catherine Bennetau-Pelissero

**Affiliations:** 1 Department Sciences and Technology for Health, Campus Carreire, University of Bordeaux, 33076 Bordeaux, France; 2 ARNA, U1212 Inserm, 5320 CNRS, Pharmacy Faculty, 33076 Bordeaux, France; 3 Centre Hospitalo Universitaire Bordeaux, USN B0-Hôpital Haut Lévêque, 33604 Pessac, France; 4 Centre Hospitalo-Universitaire Bordeaux, Laboratory of Immunology and Immunogenetics, Resources Biological Center (CRB), 33000 Bordeaux, France; 5 Bordeaux Sciences Agro, 33175 Gradignan, France

**Keywords:** phytoestrogens, soy isoflavones, enterolactone, plasma, urine, dietary inquiry, exposure assessment

## Abstract

Phytoestrogens are dietary compounds with low estrogenic activity. The two main categories in the French diet are isoflavones from pulses and enterolignans metabolized by the gut flora from various lignans found in fruits, vegetables, grains, and beverages. Isoflavones and lignans have different effects on human physiology and can antagonize each other. Comprehensive lists of phytoestrogen sources were constructed based on measurements and literature data. The 24 h and 48 h dietary recalls were proposed to the volunteers of the ISOLED cohort (NCT03421184). Urine and plasma samples from these volunteers were assayed for genistein, daidzein, equol, and enterolactone. A dietary score was constructed considering the pharmacokinetic characteristics of these compounds. Correlation analyses were applied to fluid concentrations associated with dietary scores. Pearson correlations reached 0.921 (*p* < 0.001) for urine*_IF_*, 0.900 (*p* < 0.001) for plasma*_IF_*, 0.764 (*p* < 0.001) for urine*_ENL_*, and 0.723 (*p* < 0.001) for plasma*_ENL_*. ELISAs associated with careful intake assessments proved to be good tools for phytoestrogens’ exposure estimation.

## 1. Introduction

**Figure 1:**
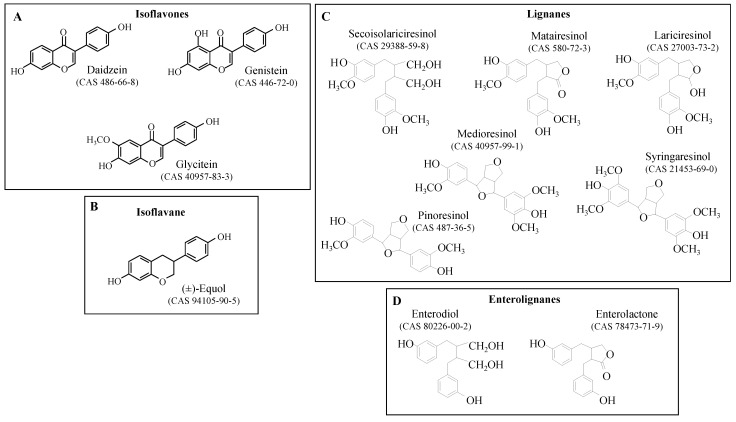
(**A**) Isoflavones, (**B**) the isoflavane metabolite equol, (**C**) the enterolactone-precursors considered in this study, and (**D**) the enterolignans known to be produced by the human gut flora.

Phytoestrogens (PHYTOs) are naturally occurring polyphenols that have endocrine effects at relevant doses [1]. Sources of estrogenic isoflavones (IF), i.e., genistein (GEN), daidzein (DAI), and glycitein (Figure 1A), in the human diet are essentially pulses including alfalfa, clover, and soy. However, nowadays, only soybean is a significant source of IFs in the French diet [2,3]. Conversely, enterolactone (ENL) is produced by the gut flora [4,5] and its precursors were shown to be numerous in the diet. Indeed, previous authors showed that besides seccoisolariciresinol di-glucoside (SECO or SDG), which is the main ENL-precursor, matairesinol (MAT), lariciresinol (LAR), pinoresinol (PINO), syringaresinol (SYR), medioresinol (MED), and some of their hydroxylated metabolites and glucosides can be metabolized into ENL [6] (see Figure 1C,D). The conversion rate was shown to vary with the individual microflora. Therefore, exposure to ENL-precursors was not necessarily associated with an estrogenic effect [7]. Moreover, ENL-precursors were shown to be present in a large panel of plant foodstuffs, including fruits, vegetables, nuts, grains, and beverages (see Table S1).

Within the fruit category, berries, plums, and citrus were consistently shown to contain significant amounts of ENL-precursors, while in the vegetables group, cabbages, squashes, soy, and legumes were consistently shown to contain significant amounts of ENL-precursors.

Therefore, while the assessment of IFs intake could simply rely on the determination of soy and “hidden soy” intakes [2,3], ENL-precursors’ intake assessment should consider many plant foodstuffs (see Table S1, and associated references).

The estrogenic effects of IF and ENL could either be beneficial, when an estrogen deficiency was diagnosed (i.e., at menopause), or adverse when estrogens were not required. This is the case in infancy, during childhood, during pregnancy, in males, or when an estrogen-dependent pathology is feared or diagnosed [8]. However, ENL was shown to have a much lower affinity for the estradiol receptors (ERs) than IFs [9]. It is also present at low doses in plasma [10] and is a selective ER modulator (SERM) whose estrogenic effect essentially relies on the activating function 2 (AF-2) domain of ERα [11]. This property probably explains the anti-proliferation effect of ENL on human breast cancer cells when present in food together with IFs in the nude athymic mice model [12] and in human population approaches [13]. Meanwhile, IFs were the subject of many debate as their in vitro estrogenic activity was found to be the same as estradiol [14]. Their proliferative effects on estrogen breast cancer cells were shown in many situations in vitro [15], in animals [16], and in humans [17]. Conversely, epidemiological studies showed that dietary soy IFs exposure can reduce the incidence of breast or colorectal cancers [18,19]. One plausible explanation would be that IFs can act as growth factors on estrogen-dependent cancer cells, while they can prevent cancerization in healthy cells. The two mechanisms may occur based on different biochemical properties of IFs.

To better understand the potential health actions of PHYTOs in the Western diet, a better estimation of the consumers’ exposure is required. Human body fluids can provide biomarkers of exposure, but their reliability should be challenged using dietary inquiries. This is what was proposed in this study where 24 h and 48 h dietary recall inquiries were proposed to the 57 volunteers enrolled so far in the ISOLED study. ISOLED analyzes the potential link between PHYTOs exposure and the occurrence of systemic lupus erythematosus (SLE) crises. SLE as an autoimmune disease which is known to exhibit a highly unbalanced sex-ratio occurrence [20]. The current hypotheses link this occurrence to genetic factors bearded by the X chromosome and to the estrogen impregnation. Indeed, SLE progression is neither observed in pre-adolescent girls nor in postmenopausal women, and the role of the environmental estrogen in SLE crises’ occurrence is under debate [21]. 

The use of body fluids as biomarker providers of PHYTOs exposure has led us to considering the substances monitored pharmacokinetics. That is why, in this study the dietary score calculated considered the delays of the appearance and disappearance of each compound in the body fluids (see Table S2A–D). The pharmacokinetic parameters were obtained in previous studies [7,22]. Correlations were calculated between fluid concentrations and dietary scores to determine the reliability of the exposure estimation. These correlation coefficients were compared to those obtained in previous studies.

## 2. Materials and Methods

### 2.1. Materials

#### 2.1.1. Volunteers

The volunteers were women included in the ISOLED clinical trial registered at clinicaltrial.gov PRS under the registration number NCT03421184, which is still in its inclusion phase. The trial was approved by the local ethics committee under the name ISOLED and the number 2017T2-29. The consort diagram flow is presented in the Supplementary Material as Figure S1. The health status of the volunteers is unknown due to the blind procedure. The protocol provided the recruitment of at least 100 premenopausal women: 30 being healthy controls without autoimmune diseases, 30 being diagnosed with SLE, and 40 having another autoimmune disease where the link with estrogens is not so obvious. This study was planned to be a first exploration, to check for the biological sample analyses and for the food inquiry feasibility. If a tendency would be detected, it was planned to open it up to a multicentric recruitment in order to find more volunteers with autoimmune diseases. The volunteers were welcomed in the Internal Medicine Service of the Haut-Lévêque Hospital in Bordeaux. Their health status was assessed by a medical questionnaire performed by a nurse and by biological analyses. The women with an autoimmune disease were already followed in the medical service and their health status was well-known. The included women were all in a premenopausal status. Their contraceptive treatment was registered. They should be healthy except for their autoimmune disease, if any. The exclusion criteria were infection with VIH, Hepatitis B and C, pregnancy or breast-feeding, menopause, and autoimmune pathology in remission or in quiescent form. All volunteers presenting a liver or a kidney disease were excluded. The health questionnaire included the gut, liver, and kidney status. They were asked to answer to a 24 h and 48 h dietary recall inquiry which was held by a nurse recruited in the medical service. No dietary recommendations were provided, and the premenopausal women recruited in the control group represented an unbiased sample of the normal French premenopausal population. The details of the open inquiries are available in the Supplementary Materials as Document 1 (SD1). Volunteers were always recruited between 9:30 am and 2:30 pm and the biological samples were all collected during this period. The time of sampling was recorded and used subsequently for the dietary score calculation. Finally, an example of the informed consent form which was translated from the French original version is available as Supplementary Document 2 (SD2).

#### 2.1.2. Sample Treatments

- Digestion and extraction of the conjugated isoflavones

The digestion of PHYTOs conjugates (glucuronides and sulfates) was performed with β-glucuronidase-aryl sulfatase from *Helix pomatia* (Roche, 10127698001), diluted (5 µL.mL^−1^) in sodium acetate buffer 0.1 M, 0.14 M EDTA, 100 UI.mL^−1^ penicillin G (SIGMA, P-3032), 0.1 mg.mL^−1^ streptomycin (SIGMA, S-6501), pH 5. The extraction of aglycone compounds was then performed using acidified ethyl-acetate (500 µL HCl 38% per L of ethyl-acetate).

- ELISA measurements

All salt reagents were from VWR-France. The protocol also involved thyroglobulin from swine (SIGMA, T1126), bovine serum albumin (EUROMEDEX, 04-100-812-C), and secondary antibody goat anti-rabbit IgG antibody-Amdex (SIGMA, RPN4301). The revelation steps required o-phenylenediamine dihydrochloride (OPD) (SIGMA, P1526). Stock solutions were prepared at 10 mg.mL^−1^ in water and stored in black vials at −22 °C. Glassware in contact with unconjugated PHYTOs was coated with Sigmacote^®^ (SIGMA, SL2). The sample, washing, and assay buffers were phosphate-buffered saline 0.01 M, 0.9% NaCl, 0.2% Tween, and 1% DMSO, pH 7.3. To obtain the saturation and antibody buffers, 1.6 mg.L^−1^ of bovine serum albumin was added to the latter. The revelation buffer was citrate-phosphate buffer 0.15 M, pH 5, with 0.05 mg of OPD.mL^−1^. The stop solution was H_2_SO_4_ 4 M. The primary antibodies were selected according to previous works [23,24,25]. They were harvested if required to avoid undesired cross-reactions with the unwanted antigen.

#### 2.1.3. Creatinine Measurements

Creatinine was measured in urine samples using the LTA Creatinine kit (LIBIOS ref. Creatinine-600). It was designed for colorimetric determination of creatinine in biological liquids without deproteinization.

### 2.2. Methods

#### 2.2.1. Dietary Inquiry and Dietary Scores

Both the 24 and 48 h dietary inquiries are open questionnaires with several items cited to avoid forgetting (see Supplementary document SD1 for details on the first 24 h recall). The inquiries were analyzed and translated into a dietary score. The latter considered the level of PHYTOs in foodstuffs that were documented either in [3] for IFs in French foodstuffs or by other investigators for ENL-precursors (see Table S1, and associated references). In those studies, the concentration values were all expressed in µg/100 g of fresh food and in aglycone equivalents. The score calculation considered the average content of PHYTOs by foodstuff combined with the occurrence of food containing PHYTOs in this category. For enterolignans-precursors, subcategories of vegetables and fruits were determined based on their average content in ENL-precursors and on common French diet habits. 

The foodstuffs considered were mainly soy-based foodstuffs and transformed foodstuffs known to contain “hidden soy”. They were soy juice, tofu/soy-based cheese, soy-food, soy-based yogurt, and transformed food (see Table S2A,B). These foodstuffs were graded according to their estimated concentrations of IFs. For instance, soy-based yogurt had a lower score than tofu or soy-based food since their IFs content was previously shown to be lower [2]. In addition, within each food category, different proportions of transformed food items were known to contain soy proteins with IFs. Indeed, within the frozen burgers category, it is known that more than 50% of items contained up to 30% of soy proteins when bought by the consumers in supermarkets. Conversely, within the frozen nuggets category, only 30% were known to contain soy proteins when bought in the same place. However, in canteens, the proportion of transformed food containing soy was shown to be higher than in equivalent food found in supermarkets. This is why when the volunteers claimed to have their meals in a canteen, an additional score was applied (see Table S2A–D).

More importantly, because both IFs and ENL were known to only be present transiently in body fluids, the scores considered the pharmacokinetic behaviours of GEN, DAI and ENL in plasma and in urine after ingestion. The pharmacokinetic parameters originated from Shinkaruk et al. study [22] for IFs and Kuijsten et al. [7] for ENL (See Table S2A–D for details). Concretely, for establishing those accurate dietary scores, six determination scales were applied to each volunteer’s consumption claims. Thus, the scales were adapted: (1) to the correlation with urine*_GEN_* concentrations, (2) to the correlation with plasma*_GEN_* concentrations, (3) to the correlation with urine*_DAI_* concentrations, (4) to the correlation with plasma*_DAI_* concentrations (5) to the correlation with urine*_ENL_* concentrations (6) to the correlation with plasma*_ENL_* concentrations (See Table S2A–D).

#### 2.2.2. ELISA Measurements in Body Fluids

ENL, GEN, DAI, and equol (EQ)—a DAI metabolite produced by the gut flora of EQ-producers-were assayed in the urine and plasma samples using specific ELISAs, as described previously [22,23]. Haptens functionalized on different carbon atoms were previously synthesized [23,24,25]. Specific polyclonal antibodies were obtained for each PHYTO bound to bovine serum albumin and the best antibodies were retained for assay development. Standard curve preparations and sample dilutions were performed in silicone-coated glass vials. The ELISAs followed a competitive procedure with an immobilized competitor, which was the homologous hapten bound to swine thyroglobulin, except for ENL [23]. The sensitivities varied between 0.08 ng/well and 0.4 ng/well, depending on the substance assayed, and intra-assay variation was always below 7% and inter-assay variation was below 17%. For the plasma samples, final dilutions varied between 1:5 and 1:20. For urine samples, the final dilutions varied between 1:5 and 1:4000 due to various levels of natural dilution of the urine samples. To take this phenomenon into account, the measured concentrations were corrected based on a creatinine excretion measurement performed on the same urine samples.

#### 2.2.3. Statistical Treatments

Each concentration value was always a mean and standard deviation of at least triplicate measurements. Due to the large cohort analyzed, the data followed a normal distribution. Therefore, the correlations between PHYTOs concentrations in fluids and dietary scores as well as their significance were calculated following the Pearson procedure. The significance was obtained by comparing the *t* value with data from Student’s significance tests at two degrees of freedom.

## 3. Results

### 3.1. Biological Fluid Measurements

#### 3.1.1. Isoflavones (IFs)

Results of IFs assayed in the urine and plasma samples of the 57 volunteers are summarized in Table 1. In urine, based on 54 samples and after correction by the creatinine excretion, the mean concentration of GEN + DAI + EQ was 8484.50 ± 61,775.14 µg.L^−1^. Three urine samples were below the detection limit and therefore were not considered for the mean, median, and standard deviation. The median concentration was 124.32 µg.L^−1^ with a minimum at 9.53 µg.L^−1^ and a maximum at 454,384.51 µg.L^−1^. Based on a urine concentration threshold at 150 µg.L^−1^, 38.60% of the volunteers were considered to have a significant IFs exposure. EQ, was detected in 26 volunteers at a urinary concentration over 20 µg.L^−1^. Additionally, EQ was undetectable in 11 urine samples and thus the parameters presented in Table 1 were obtained on 46 volunteers. This means that, based on urine detection, 56.52% of the cohort analyzed was able to significantly produce EQ. Besides, when EQ was detectable in the samples, the mean, median, minimum and maximum concentration values were 72.99 ± 219.72 ng.mL^−1^, 21.63 ng.mL^−1^, 3.37 ng.mL^−1^, and 1469.81 ng.mL^−1^, respectively.

In plasma samples, IFs concentrations (i.e., GEN + DAI + EQ) were much lower, and in 23 samples out of 57, they were not all detectable. Therefore, the summarizing data presented in Table 1 were based on 26 subjects only, for whom GEN and DAI were over the quantification limits. For IFs plasma concentrations the mean, median, minimum and maximum values were 74.95 ± 147.40 ng.mL^−1^, 21.21 ng.mL^−1^, 729.60 ng.mL^−1^ and 1.17 ng.mL^−1^, respectively. Only 14 volunteers out of 57 had a plasma concentration of GEN + DAID + EQ over 100 ng.mL^−1^. EQ was detectable in 28 plasma samples collected from the 57 volunteers, but only 12 of them showed EQ concentrations over 2 ng.mL^−1^. This corresponds to 49.12% of EQ-producers and only 29.82% of significant EQ-producers. The mean and median EQ concentrations in plasma were 4.18 ± 8.86 ng.mL^−1^ and 1.72 ng.mL^−1^, respectively. The minimum and maximum concentrations were 0.45 ng.mL^−1^ and 46.57 ng.mL^−1^, respectively.

#### 3.1.2. Enterolactone (ENL)

Results of ENL assay in the 57 urine and plasma samples are summarized in Table 1. The concentrations were corrected by the creatinine excretion to take into account the urine dilution at sampling. The mean, median, minimum and maximum urine concentrations measured were 802.53 ± 1438.85 µg.L^−1^, 304.73 µg.L^−1^., 6.76 µg.L^−1^ and 8076.13 ng.L^−1^, respectively. When dietary scores were associated with urine*_ENL_* levels, it was shown that in 6 cases (10.53%), the urine levels were low and did not reflect the dietary intake of sources of ENL-precursors. These 6 volunteers, whose urine*_ENL_* concentrations were all below 25 µg.L^−1^ (mean: 15.36 ± 7.45 µg.L^−1^) all had dietary scores around 9 (mean: 9.28 ± 2.23). Therefore, they were assigned as low-ENL-producers. Finally, 21 volunteers had urine*_ENL_* concentrations over 500 µg.L^−1^ and therefore were considered as significantly exposed to ENL-precursors.

Considering plasma*_ENL_* levels, they were much lower than the urine*_ENL_* concentrations and were generally assayed at a 1/5 and 1/10 dilutions. However thanks to the good sensitivity of the assay (mid-point of the assay curve at 2 ng.mL^−1^ of plasma), ENL was detected in all samples. The mean, median, minimum and maximum plasma concentrations were 17.89 ± 11.71 ng.mL^−1^, 13.68 ng.mL^−1^, 1.94 ng.mL^−1^, and 57.165 ng.mL^−1^, respectively. Based on ENL plasma levels over 15 ng/mL^−1^, 43.86% of the volunteers could be considered as consumers significantly exposed to ENL. In addition, 4 volunteers out of 57 had low ENL plasma levels (<10 ng.mL^−1^) despite a dietary score over 10. They were considered as low-ENL-producers and represented 7.01% of the population.

### 3.2. Dietary Inquiry and Scores

A summary of the results obtained on the dietary scores is provided in Table 2. Briefly, the urine*_ENL_* mean dietary score was 12.11 ± 5.94, and the median was close to the mean and equal to 11.40. The minimum and maximum score were 3.20 and 42.80, respectively. Considering urine*_enl_*, 28.07% of the analyzed population was considered high-consumers of ENL-precursors, i.e., with a dietary score over 15. For the plasma*_enl_* dietary scores, the mean, median, minimum and maximum dietary score were 12.38 ± 5.57, 11.73, 3.2 and 38.35 respectively. Therefore, the median was close to the mean. Based on a dietary score over 13, 40.35% of the volunteers were considered exposed to significant amounts of lignans per day.

For IFs, the results obtained on the urine*_IFs_* dietary scores analysis can be found in Table 2. The mean, median, minimum and maximum scores obtained on 54 volunteers were found to be 3.11 ± 2.85, 2.4, 0.4, and 16.90, respectively. Based on a threshold of 3, 38.60% of the volunteers were considered to have a significant IFs exposure over 48 h. Besides, in Table 2, the 26 plasma*_IFs_* dietary scores had a mean, median, minimum and maximum of 3.92 ± 3.43, 2.62, 0.63 and 16.56 respectively. Based on a urine*_IFs_* dietary score over 1, 42.11% of the volunteers were considered as significant IFs consumers.

### 3.3. Correlations between Dietary Scores and Measurements in Biological Fluids

**Figure 2:**
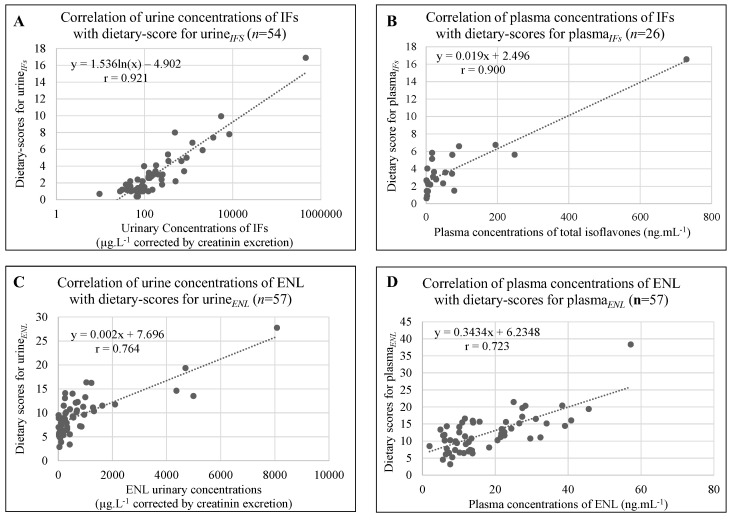
Correlations between IFs (genistein + daidzein + equol) or ENL (enterolactone) in biological fluids and their respective dietary scores.

Considering IFs, i.e., GEN + DAI + EQ urine concentrations, the Pearson correlation coefficient *r* for dietary scores reached 0.921 (*p* < 0.001) (*n* = 54). Besides, when the plasma concentrations of GEN + DAI + EQ (*n* = 26) were associated with their corresponding dietary scores, the Pearson correlation coefficient *r* was found to reach 0.900 (*p* < 0.001). The missing data correspond either to samples where IFs were undetectable or at the limit of detection or to scores which were only based on transformed foodstuff in which the occurrence of soy was not systematic. The correlations are presented in Figure 2A,B for the urine and the plasma concentrations, respectively.

The correlations between urine concentrations of GEN and DAI considered individually with their respective dietary scores of urine*_GEN_* and urine*_DAI_* are available in Figure S2A,B. Briefly, *r* for urine*_GEN_* was 0.885 and *r* for urine*_DAI_* was 0.919. In addition, correlations between plasma concentrations of GEN and DAI considered individually with their respective dietary scores are available in Figures S3A,B. Indeed, *r* for plasma*_GEN_* and plasma*_DAI_* were 0.921 and 0.846, respectively.

Considering urine*_ENL_* and plasma*_ENL_* dietary scores (*n* = 57), the Pearson correlation coefficient *r* were found to be 0.764 (*p* < 0.001), and 72.3 (*p* < 0.001), respectively. The correlation graphs are presented in Figure 2C,D.

## 4. Discussion

### 4.1. Concentrations of PHYTOs in the Body Fluids of French Volunteers

This study is the first in which IFs and ENL have been assayed in biological fluids from French volunteers following their own habitual diet. Even though these data cannot be strictly compared to previous data from a French population, they can be compared between them and with previous data obtained on Western populations. All were obtained after enzymatic hydrolysis and were expressed in aglycone equivalent.

#### 4.1.1. IFs Concentrations 

Table 1 and Table 2 showed that the standard deviation around the mean and the differences between means and medians were huge. This indicated that there was a large variability in IFs intake and consequently in measured urine and plasma concentrations. Such an observation was already previously made [2,26] by Van der Velpen and co-workers, who considered that body fluids’ concentrations of IFs could not be used to assess dietary intakes of IFs. However, the opposite was concluded in this study, possibly because one of the main variability factors, i.e., the pharmacokinetics of the ingested IFs, was taken into consideration. Moreover, only 3 urine samples among 57 were below the quantification limit of IFs. Therefore, this showed that the vast majority (95%) of the volunteers consumed IFs in the past 72 h. Details on GEN and DAI scores can be obtained from Table S2. Besides, one vegan consumer among 57 showed a chronic consumption of soy-based products and presented abnormally high concentrations of IFs in her biological fluids. Indeed, these levels reached 454,384.51 µg.L^−1^ for GEN + DAI + EQ, with 231,158.12 µg.L^−1^ and 222,912.86 µg.L^−1^ for GEN and DAI, respectively. This represents an excretion of 856.14 and 870.75 nM for GEN and DAI respectively. Besides, 38% of the volunteers were significant consumers (urine*_IFs_* > 150 µg.L^−1^ (i.e., > 0.5 µM)). Their median concentration of IFs was 347.6 µg.L^−1^ (1.3 µM). This population was recruited between 2018 and early 2022. This period partially covers the COVID-19 pandemic and the diet transition to vegan practices in France [27]. The present assessment of the exposure to IFs was in line with previous studies [2,3]. In addition, the subpopulation exposed to only low doses of IFs exhibited a median urinary excretion of 0.15 µM. Conversely, when plasma levels were examined, the highest concentration values were observed in the “high-soy” vegan consumer. For this specific volunteer, the IFs plasma level was 729.60 ng.L^−1^, with 468.15 (1.73 µM) and 253.17 ng.L^−1^ (0.99 µM) for GEN and DAI, respectively. It should be noted here that upon chronic exposure to soy IFs, GEN is usually higher in plasma than DAI due to a longer elimination half-life [28]. Looking at the significant IFs consumers, defined as persons with IFs plasma levels over 15 ng.mL^−1^, the median concentration of IFs was 66.82 ng.L^−1^, with GEN at 36.38 ng.mL^−1^ (0.135 µM) and DAI at 24.99 ng.L^−1^ (0.097 µM). It might be possible to compare these results to those previously published on Western postmenopausal women. Indeed, in [28], a study performed in the USA on post-menopausal women from the WHI cohort, the IFs plasma levels were less variable and lower than in the present study. Indeed, in [29], the mean concentration of GEN was 12.2 ± 4.3 nM, while it was 225 ± 410 nM here, when the vegan volunteer and only the detectable concentrations were included. Moreover, the mean concentration of DAI in [29] was 6.9 ± 3.6 nM, while it was 180.5 ± 241.4 nM in the present study when the vegan volunteer and only the detectable concentrations were included. When the vegan consumer was not considered, the mean for plasma*_GEN_* was 130.8 ± 136.9 nM and the mean plasma*_DAI_* was 130.0 ± 125.9 nM. When all data were included in the calculation, even those below the quantification limits and therefore being null, and without the high-soy-consumer, the mean plasma concentrations of GEN and DAI were in the range of those reported in [29] (plasma*_GEN_*: 10.09 ± 25.14 nM and plasma*_DAI_*: 9.51 ± 22.67), showing a huge variability. Finally, the plasma concentrations recorded in this study in soy consumers were between 0.1 and 1 µM, and therefore, in the low range of the IFs concentrations usually tested in vitro on cultured cells.

#### 4.1.2. Comparisons between Urine and Plasma IFs Concentrations

Looking at Table 1 and Table 2, it can be observed that the proportion of significant consumers was different when plasma or urine data were considered. This could be explained because: (1) the IFs concentrations were much lower in plasma and not always detectable using the specific ELISAs, and (2) plasma data essentially reflected a consumption of IFs which occurred within the last 24 h, while the urine data reflected an IFs exposure which occurred during the 18 to 48 previous hours. This is why for better accuracy, the dietary scores took into consideration the pharmacokinetic data of the IFs assayed. To sustain this view, the Pearson correlation coefficient *r* between IFs urinary and plasma concentrations based on the detectable plasma concentrations was only of 0.794 (*n* = 26; *p* < 0.001) (Figure S4). Such discrepancies indicated that IFs intakes were mostly occasional and most probably not performed on an everyday basis.

#### 4.1.3. Equol Production

When urine and plasma concentrations were compared, the percentage of EQ-producers was different. Indeed, based on measurements in the urine samples, 56.52% of the volunteers had detectable EQ concentrations, while based on plasma samples, 49.12% were defined as EQ-producers. These proportions were lower than those established in [30] on the same population and based on EQ measurements in hair (61.82%). This discrepancy was most probably explained because hair keeps a trace of previous exposures and metabolic transformations. However, in a previous study [28], performed on 60 postmenopausal women supplemented with 100 mg of IFs per days, the proportion of EQ-producers was 59.1%, using the same assay method. In comparison, in [29], based on 192 American women, the proportion of EQ-producers was 27.5%. Equally, in [31], based on 89 postmenopausal women, the proportion of EQ-producers was 25%. Besides, in the Isoheart study [32], which was a European multicentric clinical trial, the EQ-producers’ proportion was determined in volunteers from Germany, Denmark, the UK, and Italy. Indeed, out of 117 volunteers supplemented with 50 mg of soy IFs, the proportion of EQ producers were 50, 21.4, 25;6 and only 10% in Germany, Denmark, UK and Italy respectively. All urine samples were assayed by the AutoDelfia assay, which is a fluorescent immunoassay. Finally, it can be said that the proportion of EQ-producers varies between 20% and 60% in Europe depending on the number of assays and the method.

#### 4.1.4. The Case of the Heavy Consumer of Soybean

Heavy consumers of soybean are not rare [33,34], but usually their IFs concentrations in body fluids are not reported. Here, thanks to the 24 and 48 h dietary recalls, it was possible to link soy-food intake to body fluid concentrations. The high-soy-consumer was a healthy young vegan woman of 25 years-old who adopted a vegan diet 4 years ago but started eating soy and soy-based products only 1.5 years ago. She used to cook her own meals at home and used soy juice and soy cream for food preparations as well as chickpea meal to replace eggs in pies and cakes. She also frequently ate tofu and used soy-textured proteins in her recipes. In addition, she had five meals a day, with one collation in the morning and one in the afternoon. Briefly, in this study, she had soy juice (one mug) in the morning before sampling. On the evening preceding sampling, she had a portion of a vegan omelet with soy cream, soy juice, and chickpeas, as well as a tofu portion. At lunch of the previous day, she had a vegan quiche based on chickpea meal, soy juice, and soy cream. During the day, for collation, she had soy juice and cream in a home-made cake and a vegan quiche. Two days before the inquiry and sampling, she had soy juice in the morning and a soy-based vegan quiche for the evening meal. For collations, she had a portion of vegan omelet based on chickpea meal and soy juice and cream and a home-made cake based on soy juice. Therefore, her soy consumption was continuous and probably partly reflected her dietary habits. As can be deduced from Table 1 and Table 2, her plasma and urine IFs concentrations were 729.60 ng.mL^−1^ (about 1.7 µM GEN and 0.98 µM DAI) and 454,384.51 µg.L^−1^ (850 µM of GEN and DAI), respectively. Based on previous data obtained on French volunteers with the same assay technique [28,35,36], it seemed that the urine*_IFs_* concentrations were very high when considering the plasma*_IFs_* concentrations. This may indicate either a genetic predisposition to readily eliminate IFs from the blood or a kind of protection mechanism that was progressively developed with time and which would enhance the excretion of IFs when the chronic ingestion is high. This putative defense adaptation would result in the reduction of plasma*_IFs_* and of their potential physiological effect. Indeed, although this young woman never tried to be pregnant, she claimed having menstrual cycles typically lasting between 30 and 32 days.

#### 4.1.5. ENL Plasma and Urine Concentrations

Looking at Table S1, the intake of ENL-precursors compared to IFs appeared modest [3]. In addition, the plasma*_ENL_* levels were generally lower than plasma*_IFs_* but the urine excretion was generally higher. The plasma*_ENL_* concentrations were in accordance with previous studies [10,37,38]. As in these reports, the interindividual variations in plasma*_ENL_* levels were high, reflecting the habitual diet. Moreover, it appeared here, that the Western diet, based on potatoes (fried or boiled), hamburgers, pizzas, kebabs, etc., was associated with low intake of ENL-precursors and low fluids’ levels of ENL. On the other hand, diets rich in vegetables, berries, and wholegrain bread were associated with high intake of ENL-precursors and high body fluids’ concentrations of ENL. In the population inquired, both dietary profiles were encountered. Moreover, ENL appeared later than IFs in body fluids because of its colic production by the gut flora, while IFs were mainly absorbed in the duodenum and jejunum. This allowed a progressive accumulation of ENL in the urine compartment, and according to [7], ENL can be measured in human urine samples up to two days after a unique ingestion of ENL-precursors. However, plasma*_ENL_* concentrations (nM range) were much lower than those usually tested in vitro to decipher the mechanisms of action of ENL. This rose the question of the relevance of these in vitro data since the cell pathways potentially triggered by ENL in vitro could be different when the concentrations vary.

### 4.2. Discussion on Correlations

#### 4.2.1. Calculations of Dietary Scores

As can be seen in Table S2A–D, the dietary scores considered the level of food intake pondered by a coefficient based on the pharmacokinetic profiles of GEN, DAI, and ENL. For GEN and DAI, the data were from [22], and for ENL they were from [7]. Indeed, all samples were collected in the morning between 9:30 am and 2:30 pm. At that time, PHYTOs that were ingested at breakfast could not be seen in the IFs or ENL urine samples and were pondered by a factor of 0 in the urine dietary scores. Conversely, if foods containing IFs were ingested the previous evening, they were close to their maximum levels (urinary T*max* between 14 and 18 h) in the following morning urine samples. Thus, the ponderation of the IFs intake was the highest for GEN and DAI urine dietary scores calculation. Since the IFs urine levels were known to progressively decrease after 12 h, the urine*_IFs_* dietary scores decreased with time from sampling. For plasma, the IFs C*_max_* were known to occur around 8 h after intake. Therefore, C*_max_* was not reached after a soy intake at breakfast and the plasma*_IFs_* concentrations were already declining at sampling time after an IFs intake the previous evening. Thereafter, the plasma concentrations were known to progressively decrease, and thus the plasma dietary scores ponderations followed the same tendencies [22]. Finally, for ENL, the C*_max_* occurred in the urine between 24 and 48 h after the ENL-precursors intake in women [7]. Thus, the meals eaten 36 to 48 h before sampling were the main source of urine*_ENL_* concentrations and were attributed the highest ENL-urinary scores. Consequently, the highest ENL dietary scores for urine were seen for intakes occurring two days before sampling. If ENL-precursors were ingested on the previous evening, the urine*_ENL_* were not at their maximum levels and the urine scores for dietary intake were lower. Similarly, C*_max_* for plasma*_ENL_* was found to occur 18 to 24 h after intake in women [7,37,38]. Therefore, when ENL-precursors were ingested at lunch or dinner, before sampling, the ENL was not entirely passed in the plasma and the dietary scores applied to food intake were lower. It should be noted that, to our knowledge, this study is the first in which pharmacokinetic parameters were considered when addressing the correlations reliability between vegetable biomarkers in plasma and urine and dietary inquiries. As far as we know, such calculation was not performed in [39,40]. Producing such scores allowed to strongly link biomarkers in biological fluids and dietary intakes recorded over the last 48 h. Therefore, contrary to what was concluded by other authors, IFs and ENL in biological fluids could be advantageously and accurately used as biomarkers for assessing vegetables and legumes’ intakes.

#### 4.2.2. Comparisons of Correlation between Dietary Scores and IF in Urine or in Plasma

Like in [27,39,41], urine*_IFs_* concentrations were found to be 50 to 100 times higher than plasma*_IFs_*. Therefore, technically, it could be easier to use urine concentrations as biomarkers of IFs exposure. Additionally, urine sampling is not invasive, even if the conservation of urine samples may be more complicated than for plasma ones, due to potential oxidation at collection. Here, IFs plasma levels were sometimes below the detection limits, and this is why only 26 values could be used for the correlation with diet data. The sample size allowed the calculation of a Pearson coefficient of correlation between dietary scores and plasma*_IFs_* levels. Additionally, Figure S3 showed that the Pearson correlation coefficient between plasma*_DAI_* and its corresponding dietary score was lower than for plasma*_GEN_*. Briefly, for plasma*_DAI_*, *r* was 0.846 (*p* < 0.001), vs. 0.921 (*p* < 0.001) for plasma*_GEN_*. This may be because a part of DAI was transformed into EQ in some volunteers, decreasing the link between dietary records and plasma*_DAI_* levels. Another explanation, may be that the DAI plasma residence time is notably lower than that of GEN, and thus it varied more rapidly. Consequently, the scores used here, might have been of lower accuracy for DAI than for GEN due to interindividual variations.

#### 4.2.3. IFs and ENL as Dietary Biomarkers

It appeared that IFs and ENL can both be used as biomarkers of vegetables’ intakes (see Table S1). However, IFs were much more specific for a defined category of food, i.e., pulses, while ENL-precursors were present mainly in grains and grain-enriched foods, carob, berries, cabbages, or squashes (Table S1). Additionally, in the French diet, the main source of IFs was soybean, which appeared in soy-based foodstuffs and as “hidden soy” in transformed foodstuffs [2]. Therefore, IFs intake could also be a biomarker of transformed food consumption.

Considering the different occurrences of IFs and ENL-precursors in foodstuffs, two different dietary scales were constructed to record the two types of PHYTOs, (see Table S2A–D). Finally, it was not surprising to show that the Pearson correlation coefficient *r* between IFs and ENL urine concentrations was only 0.310 (*p* < 0.001) (*n* = 56), while the Pearson correlation coefficient *r* between urine*_IFs_* and urine*_ENL_* dietary scores was only 0.285 (*p* < 0.001) (*n* = 56) (Figure S5A,B). These scores were calculated excluding the highest consumer of IFs. Similarly, the correlation between plasma*_IFs_* and plasma*_ENL_* levels was low, as was the correlation between their respective dietary scores. These correlations were not sufficient for prediction (data not shown), and it could be deduced from these figures that, in the French diet, IFs intake was not significantly correlated with ENL-precursors’ intake.

#### 4.2.4. Comparisons with Other Published Data

Table 3 provides an overview of the correlations found in the literature between dietary inquiries and PHYTOs concentrations in biological fluids. The studies were performed either on spot urine samples, as in this study, or on urine samples collected at home over 12 or over 24 h. It should be expected that samples collected over a defined period would be more accurate, providing that no collection was missed during the period. This seemed to be confirmed comparing the correlation coefficients obtained by Grace et al. [39] for IFs spot urine levels, with those obtained by other authors that collected urine samples over a longer period. However, the sample collection is not the only source of uncertainty, and the dietary records could also be questionable. Indeed, for the previous correlations between dietary data and urine concentrations of IFs, details on the food items considered to assess the IFs exposure were not clearly disclosed. It might be hypothesized that those IFs intake records were not exhaustive because the part of “hidden soy” was usually underestimated [29]. This led to a poor correlation coefficient, lower than those observed in the present study. In addition, these correlation coefficients were quite variable from one study to another.

Considering plasma analyses, Table 3 only lists spot samples, although the dietary records could be different. Indeed, some authors performed dietary recall over 24 or 48 h or applied food frequency questionnaires (FFQ) to assess diet habits. Again, such data collections could be more or less accurate because they relied on claims from the volunteers and because the foodstuffs containing the substance of interest may not be known as accurately as they should be. Finally, if only Pearson’s correlation coefficients were analyzed, i.e., excluding the studies [41,42], the correlations obtained here were greater than those previously published, and they characterized a significant correlation. This shows that considering the pharmacokinetic parameters is important to establish relevant dietary scores. 

As seen in Table 3, the correlation coefficients between dietary records and urine*_ENL_* levels obtained by previous authors were not as good as those recorded here. Analyzing the previous studies, it was seen that sometimes the urine samplings were not appropriate considering the pharmacokinetic parameters of ENL. For instance, in [48], the urine collection was only organized over the previous 12 h and could not reflect the last 24 h intakes due to the urine T*_max_* of ENL. Additionally, in [39], the main uncertainty could come from the approximation made on the lignan intakes. Basically, the authors considered that dietary fibers well-reflected the ENL-precursors’ intakes. They did not consider specific distributions of ENL-precursors in certain fruits, vegetables, or grain categories, as was performed here (see Table S2C,D, based on Table S1). This may explain why the present study appeared to yield more accurate correlation coefficients. Again, contrary to the present studies, the previous analyses did not provide details on the foodstuffs recorded to assess ENL-precursor intakes. Therefore, it may be difficult to conclude on the relevancy of these dietary records.

### 4.3. Limits of the Study

#### 4.3.1. Phytoestrogens Analyzed

All PHYTOs were analyzed in their aglycone forms after enzymatic digestion by β-glucuronidase aryl-sulfatase, which hydrolyzed the glucurono- and sulfo-conjugates before liquid–liquid extraction by ethyl-acetate. Glycitein was not assayed in this study as it is known to be present only at a 10% ratio in soy-based products and it was usually undetectable in the plasma samples of volunteers. Moreover, its estrogenic activity is the lowest of the IFs series [49]. Additionally, ENL is not the only estrogenic metabolite of SDG, SECO, MAT, LAR, PINO, SYR, and MEDIO. Besides ENL, the other metabolite is enterodiol (Figure 1D), which was also shown to be slightly estrogenic but for which plasma and urine concentrations were found to be lower than ENL [7]. The lack of a specific ELISA for enterodiol and the specificity of the polyclonal antibody raised against ENL which has no significant recognition of enterodiol, explain why it was not assayed in this study. The ELISA for ENL was sensitive enough to assay ENL in plasma and was 10 times more sensitive than the IFs-ELISAs. This is most probably because it was a heterologous ELISA, using an immobilized ligand which was different from the hapten on which the specific polyclonal antibody was raised. Such a sensitivity was associated with a great specificity since the polyclonal antibody did not significantly recognize enterodiol nor other ENL-precursors [23]. However, urine samples were much easier to collect, and showing higher ENL concentrations when compared to plasmas ([7] and the present study), they could be better biomarkers of vegetable intakes.

#### 4.3.2. Accuracy of the Dietary Scores

One of the main advantages of this study is that it took into consideration the pharmacokinetic parameters, i.e., C*_max_*, T*_max_*, and T_1/2_, for each biomarker and included them in the dietary score. In addition, great attention was paid to the selection of the sources of biomarkers in the diet, to analyze the dietary records in the most relevant way. Indeed, the sources of IFs were derived from a previous exposure analysis of the French population and based on more than 12,000 foodstuffs labels assessment [2], while the selection of ENL-precursors was based on data collected from previous analytical studies by other authors and mentioned in Table S1. Contrary to some studies, six lignans were considered in this study, having been shown to be ENL-precursors. Indeed, besides SECO, which is the most popular ENL-precursor in countries from Northern Europe, this study also examined vegetables and grains containing significant concentrations (>100 µg/100 g of fresh weight) of other ENL-precursors. Likewise, flaxseeds are not usually consumed in France; thus, it was important to also consider foodstuffs containing MAT, LAR, PINO, MEDIO, and SYR. In doing so, it was possible to associate certain vegetable or fruit groups with higher ENL-intake coefficients. However, the correlation obtained between urine*_ENL_* concentrations and the urine*_ENL_* dietary score was only 0.764, while it reached 0.921 for IFs. This may be due to a lack of accurate data on the ENL-precursors’ concentrations in all foodstuffs and especially to the lack of data on the impact of food transformation (cooking, grilling, boiling, etc.) on ENL-precursors’ resulting concentrations. Plasma concentrations of ENL were detectable in all samples, but a large variability was observed that could be related mainly to cabbages, berries, and wholegrain bread. As for the urine*_ENL_* correlation, the plasma*_ENL_* correlation *r* was lower than that recorded for IFs, being only 0.723.

Besides, plasma*_IFs_* concentrations were sometimes too low to be detected, and thus the correlation was not calculated using all the samples. As mentioned previously, it was shown here that IFs exposure is highly variable in the French population [2,3]. Additionally, the non-soy-eater category represented about half of the population in [2,3], and this proportion was represented here with 26 out of 57 volunteers having detectable plasma*_IFS_* concentrations.

#### 4.3.3. Dietary Score Uncertainties

Regarding IFs intake, the larger uncertainties were due to the consumption of ultra-transformed meals. Indeed, based on previous data [2], four categories of transformed foodstuffs were identified: “Transformed++”, “Transformed+”, “Transformed”, and “Transformed–” (see Table S2A,B). The first subcategory contained transformed foodstuffs known to very frequently contain a high proportion of IFs, such as soy-containing burgers, meatballs, or stuffed vegetables, especially when consumed in canteens. On the other hand, the “Transformed–” category contained foodstuffs that definitely did not contain soy, such as dairy products, for instance. The two intermediate categories were defined according to their potential IFs content and based on the occurrence frequency of soy in such food groups. For instance, nuggets are known to contain soy only occasionally, but this frequency was higher in canteens. These ultra-transformed foodstuffs were the highest source of uncertainty when a dietary score was calculated for IFs.

#### 4.3.4. Uncertainties on Correlations

Another source of uncertainty came from the case of the vegan woman who had soy and IFs several times a day before sampling, and who exhibited abnormally high levels of IFs, GEN and DAI in her plasma and urine samples. The data collected on this person largely increased the variation around the mean of all concentrations or scores for IFs, GEN, and DAI. Nevertheless, because there are only few data published so far on biological samples collected on “high-soy-consumers”, it was decided to maintain these data for the analysis. As expected, considering this case impacted the correlation coefficients obtained. Indeed, when the urine*_IFs_* concentration values and urine*_IFs_* dietary scores of this volunteer were removed from the calculation of the Pearson correlation coefficient, *r* decreased from 0.921 (*p* < 0.001) to 0.864 (*p* < 0.001). Anyway, the latter coefficient was still significant and higher than those available in the literature. For plasma, based on 25 values only (without the “high-soy-consumer”), the correlation coefficient *r* between plasma concentrations and plasma*_IFs_* dietary scores was 0.653 (*p* < 0.001), vs. 0.900 (*p* < 0.001) when the “high-soy-consumer” was included. These changes in correlation coefficient and dispersion may be partly due to the low values recorded in some samples and to the presence of equol producers among the volunteers included so far.

## 5. Conclusions

Here, specific ELISAs for GEN, DAI, EQ, and ENL were used on plasma and urine samples collected at the hospital on premenopausal women who were either healthy volunteers or women with an autoimmune disease. No dietary intervention was performed, and this analysis reflects the current French diet. An open questionnaire inquiring the previous consumptions over the last 24 h and 48 h was proposed to the volunteers. It was shown that, when the pharmacokinetic parameters of the biomarkers were considered, quite accurate dietary scores could be calculated providing that the food intake was reasonably assessed. Knowing the IFs and the ENL-precursors’ content of foodstuffs was a prerequisite for such an approach, and accurate databases have been used to address this question. Indeed, knowing the global dietary habits of the French population helped to build the dietary questionnaires and scores, and those retained for French consumers may not be adapted to other populations. IFs concentrations in plasma and urine of a vegan woman overconsuming soy were reported here. The results showed a major excretion of IFs and addressed the question of a potential physiological protection phenomenon leading to lowering IFs plasma concentrations. This study also showed that nearly 60% of the French volunteers had significant EQ concentrations in their urine samples. Urine samples seemed to be good providers of pulses and ENL-precursors’ biomarkers. They can help to determine PHYTOs intake and to predict their health effects. Since natural estrogenic compounds present or deriving from food may be active in humans, they should be considered, while accurately counting endocrine disruptors in the environment. When estrogen-dependent diseases are encountered, the exposure to PHYTOs should be taken into account for more accurate health recommendations.

## Figures and Tables

**Table 1:** Summary of data obtained on phytoestrogens’ concentrations for the different analyses.

	Plasma*_IFs_* (*n* = 26)	Urine*_Ifs_* * (*n* = 54)	Urine*_EQ_* * (*n* = 46)	Plasma*_ENL_* (*n*= 57)	Urine*_ENL_* * (*n* = 57)
Mean (µg.L^−1^)	74.95	8484.94	72.99	17.89	802.53
Standard deviation (µg.L^−1^)	147.40	61,775.14	219.72	11.71	1438.85
Median (µg.L^−1^)	21.21	124.32	21.63	13.68	304.73
Min (µg.L^−1^)	1.17	9.53	3.37	1.94	6.76
Max (µg.L^−1^)	729.60	454,384.51	1469.81	57.165	8076.13
Producer characteristics (%)	-	-	56.52 ^a^	7.01 ^b^	10.53 ^b^
Significant consumers (%) **	29.82	38.60	-	40.35	36.84

IF: isoflavones (genistein + daidzein); EQ: equol; ENL: enterolactone. * Corrected by creatinine excretion, ** the thresholds for significant consumers’ determination are presented in the text. ^a.^ Significant EQ producers, urine concentrations > 20 µg.L^−1^. ^b.^ Low ENL producers, plasma concentration < 10 ng.mL^−1^ for dietary scores > 10, and urine concentration < 500 µg.L^−1^.

**Table 2:** Dietary scores for each phytoestrogen measured.

	Dietary Scores			
	Plasma*_IFs_* (*n* = 26)	Urine*_IFs_* (*n* = 54)	Plasma*_ENL_* (*n* = 57)	Urine*_ENL_* (*n* = 57)
Mean	3.92	3.11	12.38	12.11
Standard deviation	3.43	2.85	5.57	5.94
Median	2.62	2.40	11.73	11.40
Min	0.63	0.40	3.2	3.20
Max	16.56	16.90	38.35	42.80
Significant consumers (%) *	42.11	38.60	40.35	59.65

IFs: isoflavones (genistein + daidzein); ENL: enterolactone precursors. * The thresholds for significant consumers’ determination are in the text.

**Table 3:** Comparisons with other studies connecting PHYTOs in body fluids and estimated dietary intake of PHYTOs.

Subjects	Nature of Samples	Biomarkers	Dietary Data (mg.day^−1^)	Correlation	References
80 British volunteers	Plasma	GEN DAI	7-day food diaries	GEN: *r* = 0.80; *p* < 0.001 DAI: *r* = 0,45; *p* < 0.001	[42]
77 volunteers	Plasma	GEN DAI	FFQ	GEN: *r* = 0,53; *p* < 0.001 DAI: *r* = 0,45; *p* < 0.001	[29]
14 adults (14% men)	Plasma	IFs	24 h food record: (11.0) 24 h recall: (12.3)	IFs: *r* = 0.92; *p* < 0.001	[41]
333 volunteers	Serum	IFs	7-day food diaries	IFs *r* = 0.31; *p* < 0.001	[39]
203 male volunteers	Serum	IFs	FFQ	IFs: *r* = 0.27; *p* < 0.001	[43]
26 French women	Spot plasma	IFs	24 h and 48 h dietary recall	IFs: *r* = 0.900; *p* < 0.001 GEN: *r* = 0.921; *p* < 0.001 DAI: *r* = 0.846; *p* < 0.001	Present study
51 Japanese women 18 Caucasian women	24 h urine	GEN DAI	48 h dietary recall	GEN: *r* = 0.54; *p* < 0.001 DAI: *r* = 0.58; *p* < 0.001	[44]
360 women	2 overnight urines	IFs	Twice, 24 h recall DAI (µg): (5.0–6.4) GEN (µg): (7.3–9.3)	IFs: *r* = 0.52; *p* = 0.001 FFQ: *r* = 0.29; *p* < 0.01	[45]
284 volunteers	Spot urine	IFs	7-day food diaries	IFs *r* = 0.27; *p* < 0.001	[39]
14 adults (14% men)	24 h urine	IFs	24 h food record: (11.0) 24 h recall: (12.3)	IFs: *r* = 0.97; *p* < 0.001	[41]
26 premenopausal Canadian women	24 h urine	IFs	Habitual record 24 h recall	IFs: *r* = 0.64, *p* < 0.001 IFs: *r* = 0.54, *p* = 0.004	[40]
24 pubertal girls	12 h urine	IFs	3-day 24 h recall ISO: 3.0–13.3	lFs: *r* = 0.72; *p* < 0.001	[46]
256 premenopausal women	12 h urine	IFs	FFQ Low: 0.1–2.3 High: 49.8–74.6	IFs: *r* = 0.51; *p* < 0.001	[47]
100 healthy women	12 h urine	IFs	24 h recall	IFs: *r* = 0.460; *p* < 0.001	[48]
57 French women	Spot urine	IFs	24 and 48 h dietary recall	IFs: *r* = 0.921; *p* < 0.001 GEN: *r* = 0.885; *p* < 0.001 DAI: *r* = 0.919; *p* < 0.001	Present study
284 British women of the EPIC-Norfolk study	Spot urine	ENL	Fiber intake over 7-day recall	ENL: *r* = 0.29; *p* < 0.001	[39]
26 premenopausal Canadian women	24 h urine	ENL	Habitual record 24 h recall	ENL: *r* = 0.46, *p* = 0.02 ENL: *r* = 0.40, *p* = 0.05	[40]
100 apparently healthy Mexican women	12 h urine	ENL	24 h recall	ENL: *r* = 0.067; *p* = 0.580	[48]
57 healthy premenopausal French women	Spot urine Spot plasma	ENL	24 and 48 h dietary recalls	ENL: *r* = 0.764; *p* < 0.001 ENL: *r* = 0.723; *p* < 0.001	Present study

## Data Availability

Raw data are available upon request to the corresponding author.

## Supplementary Materials

Supporting information can be downloaded at: https://www.mdpi.com/article/10.3390/nu15040967/s1, Table S1: Sources of ENL-precursors according to previous analytical studies. Table S2: Dietary scales used to calculate dietary scores for DAI, GEN, and ENL. Table S2A: Scales for DAI and GEN associated with urine determination. Table S2B: Scales for DAI and GEN associated with plasma determination. Table S2C: Scales for ENL associated with urine determination. Table S2D: Scales for ENL associated with plasma determination. Figure S1: Consort diagram of the recruitment of volunteers at the time of the study and samples collected and inquiry completed. Figure S2: Correlation between urinary DAI and GEN and their respective dietary scores. Figure S2A: Correlation between urinary DAI concentrations and the corresponding dietary scores. Figure S2B: Correlation between urinary GEN concentrations and the corresponding dietary scores. Figure S3: Correlation between plasma DAI and GEN and their respective dietary scores. Figure S3A: Correlation between plasma DAI concentrations and the corresponding dietary scores. Figure S3B: Correlation between plasma GEN concentrations and the corresponding dietary scores. Figure S4: Correlation between urine and plasma IFs concentrations. Figure S5: Correlation between ENL and IFs data. Figure S5A: Correlation based on PHYTOs urine concentrations. Figure S5B: Correlation based on PHYTOs dietary scores. Document SD1: Open questionnaire proposed for the last 24 h recall. Document SD2: Example of the informed consent form (translated in English). References [50,51,52,53,54,55,56,57,58,59,60] are cited in the supplementary materials.
