# Supplementary material for: Validation of ELISAs for Isoflavones and Enterolactone for Phytoestrogen Intake Assessment in the French Population
Source: Nutrients. 2023 Feb 15;15(4):967. doi: 10.3390/nu15040967 (PMC9967075; doi:10.3390/nu15040967)
Supplement: Supplementary file 1 [file nutrients-15-00967-s001.zip › nutrients-2191610-supplementary.pdf]

Validation of ELISAs for isoflavones and enterolactone for phytoestrogen intake assessment in the French population

Souad Bensaada[1,2], Isabelle Raymond[1,3], Isabelle Pellegrin[1,4], Jean-François Viallard[1,3] and Bennetau-Pelissero Catherine[1,2,5,*]

1 University of Bordeaux, 33076 Bordeaux, France
2 ARNA, U1212 Inserm, 5320 CNRS, Pharmacy Faculty, 33076 Bordeaux, France
3 CHU Bordeaux, USN B0 – Hôpital Haut Lévêque – 33604 Pessac, France
4 CHU Bordeaux, Laboratory of Immunology and Immunogenetics, Resources Biological Center (CRB), France
5 Bordeaux Sciences Agro, 33175, Gradignan, France

*Corresponding author:
Catherine Bennetau-Pelissero
University of Bordeaux,
ARNA, U 1212 Inserm,
146 Léo Saignat Street,
BP12 33076 Bordeaux, France
catherine.bennetau@laposte.net

Abstract: Phytoestrogens are dietary compounds with low estrogenic activity. The two main categories in the French diet are isoflavones from pulses and enterolignans metabolized by the gut flora from various lignans found in fruits, vegetables, grains, and beverages. Isoflavones and lignans have different effects on human physiology and can antagonize each other. Comprehensive lists of phytoestrogen sources were constructed based on measurements and literature data. The 24 h and 48 h dietary recalls were proposed to the volunteers of the ISOLED cohort (NCT03421184). Urine and plasma samples from these volunteers were assayed for genistein, daidzein, equol, and enterolactone. A dietary score was constructed considering the pharmacokinetic characteristics of these compounds. Correlation analyses were applied to fluid concentrations associated with dietary scores. Pearson correlations reached 0.921 ($p < 0.001$) for urine_{IF}, 0.900 ($p < 0.001$) for plasma_{IF}, 0.764 ($p < 0.001$) for urine_{ENL}, and 0.723 ($p < 0.001$) for plasma_{ENL}. ELISAs associated with careful intake assessments proved to be good tools for phytoestrogens' exposure estimation.

Keywords: phytoestrogens; soy isoflavones; enterolactone; plasma; urine; dietary inquiry; exposure assessment

Abbreviations
DAI: Daidzein;
ED: Endocrine disruptor,
ENL: Enterolactone;
EQ: Equol;
GEN: Genistein
IFs: Isoflavones
PHYTOs: Phytoestrogens

List of supplementary data

Table S1. Sources of ENL precursors according to previous analytical studies

Table S2. Dietary scales used to calculate dietary scores for DAI, GEN and ENL. **Table S2A**, Scales for DAI and GEN associated with urine determination; **Table S2B**, Scales for DAI and GEN associated with plasma determination, **Table S2C**, Scales for ENL associated with urine determination. **Table S2D**, Scales for ENL associated with plasma determination.

Figure S1. Consort diagram of the recruitment of volunteers at the time of the study and samples collected and inquiry completed.

Figure S2. Correlation between urinary DAI and GEN and their respective dietary scores. **Fig S2A**: correlation between urinary DAI concentrations and the corresponding dietary scores. **Fig S2B**: correlation between urinary GEN concentrations and the corresponding dietary scores.

Figure S3. Correlation between plasma DAI and GEN and their respective dietary scores. **Fig S3A**: correlation between plasma DAI concentrations and the corresponding dietary scores. **Fig S3B**: correlation between plasma GEN concentrations and the corresponding dietary scores.

Figure S4. Correlation between urine and plasma IFs concentrations.

Figure S5. Correlation between ENL and IFs data. **Fig S5A**. correlation based on PHYTOs urine concentrations; **Fig S5B**. correlation based on PHYTOs dietary scores.

Supplementary Document SD1. Open questionnaire proposed for the last 24 hours recall.

Supplementary Document SD2. Example of an inform consent form (translated in English)

Table S1. Sources of ENL precursors according to previous analytical studies

Fruits & Vegetables	Secoisolariciresinol (CAS 148244-82-0)	Matairesinol (CAS 580-72-3)	Lariciresinol (CAS 27003-73-2)	Pinoresinol (CAS 487-36-5)	Syringaresinol (CAS 21453-69-0)	Medioresinol (CAS 40957-99-1)	Total	References
			µg/100g fresh weight					
**Fruits**								
Abricot	31.00	0.00	105.00	314.00	na	na	450.00	Milder et al., 2005
Apple	na	3.00	55.00	na	na	na	58.00	Penalvo et al., 2008
Apricots dry	328.3	tr	na	na	na	na	328.30	Horn-Ross et al., 2000
Apricots dry	147.60	0.60	62.10	190.10	na	na	399.10	Thompson et al., 2006
Almond	70.30	0.30	32.20	9.00	na	na	111.80	Thompson et al., 2006
Ananas	7.00	18.00	24.00	3.00	na	na	52.00	Penalvo et al., 2008
Banana	nd	17.00	na	19.00	na	na	36.00	Penalvo et al., 2008
Cherry	6.00	0,00	41.00	100.00	na	na	147.00	Milder et al., 2005
Chestnuts	201.00	13.00	na	na	na	na	214.00	Kuhnle et al. 2009
Chestnuts	172.7	0.5	7.8	5.6	na	na	186.60	Thompson et al., 2006
Chestnuts cooked	265.00	15.00	na	na	na	na	280.00	Kuhnle et al. 2009
Dates	161.00	3.00	na	na	na	na	164.00	Kuhnle et al., 2009
Dates	581.00	3.00	na	na	na	na	584.00	Kuhnle et al., 2009
Dates	156.00	2.00	na	na	na	na	158.00	Kuhnle et al., 2009
Date dry	106.2	0.3	116.9	100.2	na	na	323.60	Thompson et al., 2006
Figs	372.00	3.00	na	na	na	na	375.00	Kuhnle et al., 2009
Figs dry	113.00	1.00	na	na	na	na	114.00	Kuhnle et al., 2009
Greengage	102.00	1.00	na	na	na	na	103.00	Kunhle et al., 2009
Grenad	294.00	9.00	na	na	na	na	303.00	Kuhnle et al., 2009
Kiwi fruits	174.6	1.2	na	na	na	na	175.80	Valsta et al., 2003
Kiwi fruits	107.00	4.00	na	na	na	na	111.00	Kuhnle et al., 2009
Kiwi fruits	112.00	0.00	17.00	0.00	na	na	129.00	Milder et al., 2005
Kiwi fruits	106.00	na	20.00	13.00	na	na	139.00	Penalvo et al., 2008
Lingonberries	140.00	nd	19.00	586.00	na	na	745.00	Nurmi et al., 2010
Loquat	0.28	0.00	6.9	179.99	23.43	9.00	219.60	Moreno-Franco et al., 2011
Nashi	7.00	na	21.00	na	na	na	28.00	Penalvo et al., 2008
Nectarine	18.00	0.00	41.00	131.00	na	na	190.00	Milder et al., 2005
Peaches	11.00	na	38.00	83.00	na	na	132.00	Penalvo et al., 2008
Peaches	27.00	0.00	80.00	186.00	na	na	293.00	Milder et al., 2005
Peaches	28.00	nd	na	na	na	na	28.00	Horn-Ross et al., 2000
Pears	2.00	na	34.00	2.00	na	na	38.00	Penalvo et al., 2008
Pears	9.90	0.70	na	na	na	na	10.60	Valsta et al., 2003
Pears	4.00	0.00	155.00	34.00	na	na	193.00	Milder et al., 2005

Fruits & Vegetables	Secoisolariciresinol (CAS 148244-82-0)	Matairesinol (CAS 580-72-3)	Lariciresinol (CAS 27003-73-2)	Pinoresinol (CAS 487-36-5) μg/100g fresh weight	Syringaresinol (CAS 21453-69-0)	Medioresinol (CAS 40957-99-1)	Total	References
**Fruits**								
Plums	16.00	na	20.00	42.00	na	na	78.00	Penalvo et al., 2008
Plums cooked	352.00	4.00	na	na	na	na	356.00	Kuhnle et al., 2009
Plums cooked & dried	105.00	<1	na	na	na	na	105.00	Kuhnle et al., 2009
Plums half dried	278.00	3.00	na	na	na	na	281.00	Kuhnle et al., 2009
Plums dried	103.8	0.2	2.1	71.5	na	na	177.60	Thompson et al., 2006
Plums dried yellow	150.00	<1	na	na	na	na	150.00	Kuhnle et al., 2009
Plums	76,00	tr	na	na	na	na	76.00	Horn-Ross et al., 2000
Raisins	tr	52.00	na	na	na	na	52.00	Horn-Ross et al., 2000
Raisins	2.00	1.3	na	na	na	na	3.30	Valsta et al., 2003
Raisins white	9.00	19.00	153.00	0.00	na	na	181.00	Milder et al., 2005
Raisins blue	8.00	18.00	118.00	0.00	na	na	144.00	Milder et al., 2005
Rosehip berry	78.8	2.00	na	na	na	na	81.00	Valsta et al., 2003
**Citrus**								
Bitter orange	26.00	na	192.00	654.00	na	na	872.00	Penalvo et al., 2008
Grapefruits	nd	tr	na	na	na	na	0.00	Horn-Ross et al., 2000
Grapefruits	26.30	0.00	na	na	na	na	26.30	Valsta et al., 2003
Grapefruits	9.00	2.00	95.00	45.00	na	na	151.00	Milder et al., 2005
Navelle orange	14.00	na	128.00	24.00	na	na	166.00	Penalvo et al., 2008
Orange (juice)	tr	tr	na	na	na	na	0.00	Horn-Ross et al., 2000
Valencia orange	56.00	na	193.00	51.00	na	na	300.00	Penalvo et al., 2008
**Vegetables**								
Advocate	47.00	6.00	31.00	272.00	na	na	356.00	Penalvo et al., 2008
**Artichoke**	**171.45**	**0.00**	**153.76**	**3479.71**	**21.89**	**56.65**	**3883.47**	Moreno-Franco et al., 2011
Asparagus	743.00	14.00	92.00	na	na	na	849.00	Penalvo et al., 2008
Asparagus green	78.30	3.40	na	na	na	na	81.70	Valsta et al., 2003
Asparagus white	28.70	0.60	na	na	na	na	29.30	Valsta et al., 2003
Asparagus	68.00	tr	na	na	na	na	68.00	Horn-Ross et al., 2000
Bamboo sprouts	38.00	na	na	na	na	na	38.00	Penalvo et al., 2008
Beet	8.00	0.00	na	na	na	na	8.00	Valsta et al., 2003
Carrots	38.00	tr	na	na	na	na	38.00	Horn-Ross et al., 2000
Carrots	18.00	0.00	64.00	37.00	na	na	119.00	Milder et al., 2005
Carrots cooked	107.00	3.00	na	na	na	na	110.00	Kuhnle et al., 2009
Carrots in can	47.00	1.00	na	na	na	na	48.00	Kuhnle et al., 2009
Celery	12.00	na	16.00	43.00	na	na	71.00	Penalvo et al., 2008
Celery	9.90	0.00	na	na	na	na	9.90	Valsta et al., 2003

Fruits & Vegetables	Secoisolariciresinol (CAS 148244-82-0)	Matairesinol (CAS 580-72-3)	Lariciresinol (CAS 27003-73-2)	Pinoresinol (CAS 487-36-5)	Syringaresinol (CAS 21453-69-0)	Medioresinol (CAS 40957-99-1)	Total	References
				µg/100g fresh weight				
**Vegetables**								
Cherry tomatoes	17.00	na	43.00	11.00	na	na	71.00	Penalvo et al., 2008
Cherry tomatoes	16.00	na	38.00	19.00	na	na	73.00	Penalvo et al., 2008
Corn	125.00	21.00	69.00	33.00	220.00	nd	468.00	Smeds et al., 2007
Corn (dwarf)	na	na	25.00	16.00	na	na	41.00	Penalvo et al., 2008
Corn (dwarf)	7.00	na	14.00	na	na	na	21.00	Penalvo et al., 2008
Corn (grilled)	14.46	0.00	15.33	0.00	331.55	3.01	364.35	Moreno-Franco et al., 2011
Eggplant	8.00	na	40.00	51.00	na	na	99.00	Penalvo et al., 2008
Leek	11.80	0.00	na	na	na	na	11.80	Valsta et al., 2003
Onions, leek, garlic	34.00	0.00	153.00	100.00	na	na	287.00	Tetens et al., 2013
Pasta Cooked	4.00	0.00	7.00	5.00	na	na	16.00	Tetens et al., 2013
Potato, chips	<1.00	2.00	na	na	na	na	3.00	Kuhnle et al., 2009b
Potato, chips (Oven)	3.00	1.00	na	na	na	na	4.00	Kuhnle et al., 2009b
Potato, crisps	14.00	2.00	na	na	na	na	17.00	Kuhnle et al., 2009b
Potato, (Boiled)	<1.00	<1.00	na	na	na	na	1.00	Kuhnle et al., 2009b
Potato, (Boiled)	<1.00	<1.00	na	na	na	na	<1.00	Kuhnle et al., 2009b
Potato, mashed, instar	1.00	<1.00	na	na	na	na	1.00	Kuhnle et al., 2009b
Potatoes	4.00	2.00	0.00	10.00	na	na	16.00	Tetens et al., 2013
Salad (Letuce)	105.60	0.6	na	na	na	na	106.20	Valsta et al., 2003
Spinach	8.00	na	110.00	31.00	na	na	149.00	Penalvo et al., 2008
Spinach	13.00	1.00	79.00	15.00	na	na	108.00	Penalvo et al., 2008
Sweet potatoes	65.00	2.00	79.00	16.00	na	na	162.00	Penalvo et al., 2008
Sweet potatoes	122.00	<1	na	na	na	na	122.00	Kuhnle et al., 2009
Sweet potatoes cooke	132.00	<1	na	na	na	na	132.00	Kuhnle et al., 2009
Sweet potatoes	nd	41.00	na	na	na	na	41.00	Horn-Ross et al., 2000
Tomato	3.00	na	11.00	12.00	na	na	26.00	Penalvo et al., 2008
Tomato (paste)	9.00	0.00	107.00	70.00	na	na	186.00	Milder et al., 2005
**Mushrooms**								
Ceps	0.60	0.30	na	na	na	na	0.90	Valsta et al., 2003
Chanterels	3.90	7.30	na	na	na	na	11.20	Valsta et al., 2003
Black trumpets	1.30	0.10	na	na	na	na	1.40	Valsta et al., 2003
Shiitake	0.20	0.00	na	na	na	na	0.20	Valsta et al., 2003

Fruits & Vegetables	Secoisolariciresinol (CAS 148244-82-0)	Matairesinol (CAS 580-72-3)	Lariciresinol (CAS 27003-73-2)	Pinoresinol (CAS 487-36-5) µg/100g fresh weight	Syringaresinol (CAS 21453-69-0)	Medioresinol (CAS 40957-99-1)	Total	References
**Berries**								
Blackberries	12.00	nd	166.00	54.00	na	na	232.00	Nurmi et al., 2010
Blackberries	220.00	nd	na	na	na	na	220.00	Kuhnle et al., 2009
Cranberry	193.00	nd	69.00	122.00	na	na	384.00	Nurmi et al., 2010
Currants	119.00	2.00	na	na	na	na	121.00	Kuhnle et al., 2009
Strawberries	51.00	na	33.00	21.00	na	na	105.00	Penalvo et al., 2008
Strawberries	5.00	nd	117.00	212.00	na	na	334.00	Nurmi et al., 2010
Strawberries	5.00	0.00	117.00	212.00	na	na	334.00	Milder et al., 2005
Strawberry	5.00	0.00	117.00	212.00	na	na	334.00	Tetens et al., 2013
Strawberries	61.37	18.3	134.00	5.21	60.83	3.89	284.49	Moreno-Franco et al., 2011
**Oil seeds**								
Olive	55.90	2.70	na	na	na	na	58.60	Valsta et al., 2003
Olive oil	1.00	0.30	1.70	139.70	na	na	142.70	Thompson et al., 2006
Olive oil extra virgin	0.00	0.00	4.00	243.00	na	na	247.00	Milder et al., 2005
Olive oil standard	0.00	0.00	5.00	101.00	na	na	106.00	Milder et al., 2005
Sunflower seeds	128.00	nd	na	na	na	na	128.00	Horn-Ross et al., 2000
Sunflower seeds	26.20	0.50	149.70	33.90	na	na	210.30	Thompson et al., 2006
Sunflower seeds	106.00	3.00	na	na	na	na	109.00	Kuhnle et al., 2008
Sunflower seeds	53.00	0.00	671.00	167.00	na	na	891.00	Milder et al., 2005
**Nuts**								
Almonds	159.00	24.00	233.00	208.00	40.00	nd	664.00	Smeds et al., 2007
Brazil nuts	770.00	12.00	na	na	na	na	782.00	Kuhnle et al., 2008
**Carob**	**1266.51**	**108.41**	**1770.74**	**294.99**	**12965.7**	**336.08**	**16742.43**	Moreno-Franco et al., 2011
Cashew nuts	165.00	5.00	na	na	na	na	170.00	Kuhnle et al., 2008
Cashew nuts	37.50	0.30	60.50	1.10	na	na	99.40	Thompson et al., 2006
Cashew nuts	133.00	0.00	496.00	0.00	na	na	629.00	Milder et al., 2005
Cashew nuts	316.00	55.00	307.00	19.00	26.00	nd	723.00	Smeds et al., 2007
Coconuts (powder)	34.00	0.00	na	na	na	na	34.00	Valsta et al., 2003
Peanuts	116.00	19.00	98.00	32.00	81.00	nd	346.00	Smeds et al., 2007
Peanuts grilled salted	256.00	17.00	na	na	na	na	273.00	Kuhnle et al., 2008
Peanuts grilled salted	53.00	0.00	41.00	0.00	na	na	94.00	Milder et al., 2005
Pistachio	44.60	0.10	123.00	31.20	na	na	198.90	Thompson et al., 2006
Walnuts	140.00	5.00	na	na	na	na	145.00	Kuhnle et al., 2008
Walnuts	99.00	60.00	47.00	34.00	24.00	nd	264.00	Smeds et al., 2007

Fruits & Vegetables	Secoisolariciresinol (CAS 148244-82-0)	Matairesinol (CAS 580-72-3)	Lariciresinol (CAS 27003-73-2)	Pinoresinol (CAS 487-36-5) µg/100g fresh weight	Syringaresinol (CAS 21453-69-0)	Medioresinol (CAS 40957-99-1)	Total	References
**Legumes**								
Alafalfa sprouts	tr	nd	na	na	na	na	0.00	Horn-Ross et al., 2000
Beans	66.00	na	128.00	31.00	na	na	225.00	Penalvo et al., 2008
Beans	58.00	na	144.00	37.00	na	na	239.00	Penalvo et al., 2008
Beans	29.00	0.00	220.00	24.00	na	na	273.00	Milder et al., 2005
Beans (red)	nd	nd	na	na	na	na	0.00	Horn-Ross et al., 2000
Beans (red)	240.02	9.00	422.45	20.85	16.98	2.83	712.13	Moreno-Franco et al., 2011
Beans (red)	240.39	34.89	319.23	24.67	57.34	4.77	681.29	Moreno-Franco et al., 2011
Beans (red)	153.00	19.46	218.24	38.00	186.57	20.81	636.08	Moreno-Franco et al., 2011
Beans (red)	139.15	29.00	92.79	48.86	69.00	19.12	397.92	Moreno-Franco et al., 2011
Beans (red)	92.99	21.03	88.27	47.39	31.53	15.9	297.11	Moreno-Franco et al., 2011
Beans (red)	167.85	11.70	32.00	2.90	72.00	2.41	288.86	Moreno-Franco et al., 2011
Beans (red)	140.47	10.89	248.01	33.43	157.00	19.8	609.60	Moreno-Franco et al., 2011
Broad beans	240.00	34.89	319.23	24.67	57.34	4.77	681.29	Moreno-Franco et al., 2011
Clover sprouts	nd	nd	na	na	na	na	0.00	Horn-Ross et a., 2000
Humus	2.50	15.50	123.70	834.70	na	na	976.40	Thompson et al., 2006
Lentils	1.38	245.17	233.24	86.93	196.63	17.00	780.35	Moreno-Franco et al., 2011
Mungo beans	289.00	nd	na	na	na	na	289.00	Kuhnle et al., 2009
Mungo sprouts	97.00	0.1	18.5	13.1	na	na	128.70	Thompson et al., 2006
Mungo sprouts	82.00	1.00	32.00	33.00	na	na	148.00	Penalvo et al., 2008
Mungo sprouts	tr	tr	na	na	na	na	0.00	Horn-Ross et al., 2000
Peas	129.00	na	9.00	6.00	na	na	144.00	Penalvo et al., 2008
Peas	na	na	59.00	50.00	na	na	109.00	Penalvo et al., 2008
Peas	2.73	6.79	150.59	79.81	175.36	24.20	439.48	Moreno-Franco et al., 2011
Peas	1.00	98.82	83.73	7.11	1.82	0.00	192.63	Moreno-Franco et al., 2011
Peas	2.61	87.75	133.75	8.00	1.81	0.00	234.14	Moreno-Franco et al., 2011
Soy meal	79.10	na	99.60	88.70	na	na	267.40	Thompson et al., 2006
Soy meal	337.00	9.00	na	na	na	na	346.00	Kuhnle et al., 2009
Tofu	18.00	0.00	61.00	61.00	na	na	140.00	Milder et al., 2005
**Cucurbitaceae**								
Cucumber	41.00	na	65.00	na	na	na	106.00	Penalvo et al., 2008
Pumkin	20.00	na	29.00	na	na	na	49.00	Penalvo et al., 2008
Pumkin	153.00	<1	na	na	na	na	153.00	Kuhnle et al. 2009
Pumkin	29.00	3.00	58.00	8.00	na	na	98.00	Penalvo et al., 2008
Pumkin seeds	510.00	11.00	na	na	na	na	521.00	Kuhnle et al., 2008
Spanish melon	23.00	na	69.00	12.00	na	na	104.00	Penalvo et al., 2008
Water melon	5.00	na	67.00	5.00	na	na	77.00	Penalvo et al., 2008
Witer squash	13.40	0.10	91.10	8.80	na	na	113.40	Thompson et al., 2006
Zucchini	18.00	0.00	64.00	37.00	na	na	119.00	Milder et al., 2005

Fruits & Vegetables	Secoisolariciresinol (CAS 148244-82-0)	Matairesinol (CAS 580-72-3)	Lariciresinol (CAS 27003-73-2)	Pinoresinol (CAS 487-36-5)	Syringaresinol (CAS 21453-69-0)	Medioresinol (CAS 40957-99-1)	Total	References
			μg/100g fresh weight					
**Brassicaceae**								
Broccoli	38.00	0.00	972.00	315.00	na	na	1325.00	Milder et al., 2005
Broccoli	tr	nd	na	na	na	na	0.00	Horn-Ross et al., 2000
Brussels sprouts	30.00	nd	na	na	na	na	30.00	Horn-Ross et al., 2000
Brussels sprouts	21.00	0.6	na	na	na	na	21.60	Valsta et al., 2003
Brussels sprouts	34.00	0.00	493.00	220.00	na	na	747.00	Milder et al., 2005
Cabbage red	9.00	0.00	178.00	90.00	na	na	277.00	Milder et al., 2005
Cabbage white	8.00	0.00	212.00	568.00	na	na	788.00	Milder et al., 2005
Cauliflower	91.00	na	36.00	85.00	na	na	212.00	Penalvo et al., 2008
Cauliflower	4.00	0.00	124.00	58.00	na	na	186.00	Milder et al., 2005
Cabbage Kale	16.00	12.00	599.00	1691.00	na	na	2321.00	Milder et al., 2005
Horseradish	14.00	0.00	na	na	na	na	14.00	Valsta et al., 2003
Horseradish	37.00	49.00	219.00	16.00	na	na	321.00	Penalvo et al., 2008
Radish	na	na	29.00	43.00	na	na	72.00	Penalvo et al., 2008
Radish	1.00	15.00	na	40.00	na	na	56.00	Penalvo et al., 2008
Rutabaga	3.70	0.60	na	na	na	na	4.30	Valsta et al., 2003
Turnips	6.30	0.00	na	na	na	na	6.30	Valsta et al., 2003
**Bread grains & meals**								
Barley	42.00	42.00	133.00	71.00	140.00	22.00	450.00	Smeds et al., 2007
Buckwheat	350.00	39.00	505.00	63.00	145.00	16.00	1118.00	Smeds et al., 2007
Crackers	38.71	5.26	79.85	244.17	107.02	6.44	481.45	Moreno-Franco et al., 2011
Croissant	33.10	0.00	9.99	0.00	73.57	1.89	118.55	Moreno-Franco et al., 2011
Cereal bar	25.53	1.42	47.95	142.32	106.91	4.90	469.61	Moreno-Franco et al., 2011
Cereal chocolate	22.25	1.90	31.71	41.29	145.04	6.03	248.22	Moreno-Franco et al., 2011
Dhurra	13.00	1.90	3.80	33.00	90.00	nd	141.70	Smeds et al., 2007
**Flax seeds**	**375321.9**	**153.3**	**2807.5**	**729.6**	**na**	**na**	**379012.30**	Thompson et al., 2006
**Flax seeds**	**294210.00**	**553.00**	**3041.00**	**3324.00**	**na**	**na**	**301128.00**	Milder et al., 2005
French baguette	8.56	0.00	14.21	11.47	85.83	2.86	122.93	Moreno-Franco et al., 2011
**Grain bread**	**6163.00**	**19.00**	**185.00**	**377.00**	**na**	**na**	**6744.00**	Milder et al., 2005
**linseeds**	**165759**	**529**	**1780**	**871**	**48**	**nd**	**168987.00**	Smeds et al., 2007
**Linseed bread**	**7208.30**	**1.20**	**9.80**	**4.10**	**na**	**na**	**7223.40**	Thompson et al., 2006
**Linseed bread**	**11845.00**	**26.00**	**220.00**	**283.00**	**nq**	**na**	**12474.00**	Milder et al., 2005
Millet	80.00	82.00	34.00	216.00	96.00	12.00	520.00	Smeds et al., 2007
Muesli 1	17.00	0.00	250.00	497.00	na	na	764.00	Milder et al., 2005
Muesli 2	13.00	0.00	120.00	210.00	na	na	343.00	Milder et al., 2005
Muesli 3	17.00	0.00	63.00	129.00	na	na	209.00	Milder et al., 2005
Oat	90.00	440.00	766.00	567.00	297.00	112.00	2272.00	Smeds et al., 2007
Quinoa	30.00	15.00	125.00	54.00	180.00	nd	404.00	Smeds et al., 2007

Fruits & Vegetables	Secoisolariciresinol (CAS 148244-82-0)	Matairesinol (CAS 580-72-3)	Lariciresinol (CAS 27003-73-2)	Pinoresinol (CAS 487-36-5)	Syringaresinol (CAS 21453-69-0)	Medioresinol (CAS 40957-99-1)	Total	References
				µg/100g fresh weight				
**Bread grains & meals**								
Rice (Japanese)	39.00	nd	757.00	503.00	967.00	74.00	2340.00	Smeds et al., 2007
Rice (wild)	46.00	1.22	55.00	194.00	1116.00	92.00	1504.22	Smeds et al., 2007
Rice (red)	66.00	87.00	71.00	60.00	160.00	24.00	468.00	Smeds et al., 2007
Rice (brown)	20.00	1.60	153.00	66.00	99.00	27.00	366.60	Smeds et al., 2007
Rice bread	33.00	4.00	47.00	44.00	na	na	128.00	Penalvo et al., 2008
Rice bread	7.00	1.00	18.00	16.00	na	na	42.00	Penalvo et al., 2008
Refined bread	17.00	0.00	38.00	28.00	na	na	83.00	Milder et al., 2005
Rye	462.00	729.00	1503.00	1547.00	3540.00	585.00	8639.00	Smeds et al., 2007
Rye bread	122.00	0.20	11.40	9.40	na	na	143.00	Thompson et al., 2006
Rye bread	16.00	14.00	122.00	172.00	na	na	324.00	Milder et al., 2005
Rye bread	15.00	12.00	111.00	163.00	na	na	301.00	Milder et al., 2005
Rye bread	17.00	0.00	73.00	33.00	na	na	123.00	Milder et al., 2005
Sesame seed	7.30	123.10	1052.40	6814.50	na	na	7996.80	Thompson et al., 2006
**Sesame seed**	**66.00**	**481.00**	**9470.00**	**29331.00**	**na**	**na**	**39348.00**	Milder et al., 2005
**Sesame seeds**	**240.00**	**1137.00**	**13060.00**	**47136.00**	**205.00**	**4153.00**	**65931.00**	Smeds et al., 2007
Spelt wheat	155.00	8.60	583.00	494.00	1126.00	291.00	2657.60	Smeds et al., 2007
Tea biscuits	40.42	0.00	12.26	6.45	70.38	2.02	131.53	Moreno-Franco et al., 2011
Triticale	165.00	34.00	402.00	518.00	899.00	165.00	2183.00	Smeds et al., 2007
Wheat	868.00	410.00	672.00	138.00	882.00	232.00	3202.00	Smeds et al., 2007
White bread	7.44	0.00	14.75	9.02	108.6	2.87	142.68	Moreno-Franco et al., 2011
White bread	0.00	0.00	11.00	7.00	na	na	18.00	Milder et al., 2005
Whole bread	19.44	0.00	32.48	23.37	195.59	6.28	277.16	Moreno-Franco et al., 2011
Whole bread	15.00	0.00	73.00	33.00	na	na	121.00	Milder et al., 2005
Whole weat meal	31.00	0.00	140.00	38.00	na	na	209.00	Milder et al., 2005
**Condiments**								
Amaranth	98.00	33.00	45.00	53.00	47.00	114.00	390.00	Smeds et al., 2007
Basilic	546.00	1.90	na	na	na	na	546.00	Valsta et al., 2003
Capres	44.50	15.10	na	na	na	na	59.60	Valsta et al., 2003
Garlic	55.00	na	84.00	45.00	na	na	184.00	Penalvo et al., 2008
Garlic	93.00	4.00	na	na	na	na	97.00	Kuhnle et al., 2009
Garlic	50.00	0.00	286.00	200.00	na	na	536.00	Milder et al., 2005
Garlic	27.00	37.00	na	na	na	na	64.00	Horn-Ross et al., 2000
Ginger	na	16.00	na	15.00	na	na	31.00	Penalvo et al., 2008
Ginger	21.30	0.00	na	na	na	na	21.30	Valsta et al., 2003
Green pepper	5.00	na	73.00	6.00	na	na	84.00	Penalvo et al., 2008
Green pepper	7.00	0.00	106.00	1.00	na	na	114.00	Milder et al., 2005
Origano (dry)	44.40	1.00	na	na	na	na	45.40	Valsta et al., 2003
Paprika	8.00	79.00	2.00	na	na	na	89.00	Penalvo et al., 2008
Red pepper	9.00	na	73.00	1.00	na	na	83.00	Penalvo et al., 2008
Red pepper	7.00	0.00	106.00	1.00	na	na	114.00	Milder et al., 2005

Fruits & Vegetables		Secoisolariciresinol (CAS 148244-82-0)	Matairesinol (CAS 580-72-3)	Lariciresinol (CAS 27003-73-2)	Pinoresinol (CAS 487-36-5)	Syringaresinol (CAS 21453-69-0)	Medioresinol (CAS 40957-99-1)	Total	References
				µg/100g fresh weight					
**Beverages**									
	Beer (mix)	0.5	0.0	7.6	17.4	na	na	25.5	Tetens et al., 2013
	Coffee caspules	17.00	nd	5.30		na	na	22.30	Angeloni et al., 2020
	Coffee instant	862.00	58.00	na	na	na	na	920.00	Kuhnle et al., 2008
	Coffee instant decaf.	610.00	32.00	na	na	na	na	642.00	Kuhnle et al., 2008
	Coffee (mix)	12.7	0.35	11.1	0.95	na	na	25.00	Tetens et al., 2013
	Coffee powder	13.35	nd	4.25	na	na	na	17.60	Angeloni et al., 2020
	Coffee grain	14.34	nd	5.31	na	na	na	19.65	Angeloni et al., 2020
	Chamomile	440.67	0.00	0.00	2798.51	2444.09	276.00	5959.27	Moreno-Franco et al., 2011
	Dark chocolate	0.00	0.00	20.00	23.00	na	na	43.00	Tetens et al., 2013
	Cocoa powder	8.00	0.00	26.00	26.00	na	na	60.00	Tetens et al., 2013
	Tea (mix black/green)	9.00	1.6	24.8	23.2	na	na	58.4	Tetens et al., 2013
	Wine	25.60	3.25	5.50		3.60	61.54	99.49	Nurmi et al.; 2003
	Red Wine	51.80	0.10	13.00	0.80	na	na	65.70	Thompson et al., 2006
	Wine (mix)	33.3	5.3	10.4	6.8	na	na	55.7	Tetens et al., 2013

References.

Angeloni et al. Quantification of lignans in 30 ground coffee samples and evaluation of theirs extraction yield in espresso coffee by HPLC-MS/MS triple quadrupole. *Int J Food Sci Nutr* **2020**, *71(2)*, 193–200 doi: 10.1080/09637486.2019.1624693

Horn-Ross et al. Sources of phytoestrogen exposure among non-Asian women in California, USA. *Cancer Causes Control* **2000**, *11(4)*, 299-302. doi: 10.1023/a:1008968003575.

Kuhnle et al. Phytoestrogen content of beverages, nuts, seeds, and oils. *J Agric Food Chem*. **2008**, *56(16)*, 7311-7315. doi: 10.1021/jf801534g.

Kuhnle et al. Phytoestrogen content of cereals and cereal-based foods consumed in the UK. *Nutr Cancer*. **2009**, *61(3)*, 302-309. doi: 10.1080/01635580802567141.

Kuhnle et al. Phytoestrogen content of fruits and vegetables commonly consumed in the UK based on LC–MS and 13C-labelled standards. *Food Chem* **2009**, *116*, 542-554. doi:10.1016/j.foodchem.2009.03.002

Milder et al. Intake of the Plant Lignans Secoisolariciresinol, Matairesinol, Lariciresinol, and Pinoresinol in Dutch Men and Women. *J Nutr* **2005**, *135(5)*, 1202-1207. doi: 10.1093/jn/135.5.1202.

Moreno-Franco et al. Dietary Alkylresorcinols and Lignans in the Spanish Diet: Development of the Alignia Database. *J Agric Food Chem* **2011**, *59(18)*, 9827-9834. doi: 10.1021/jf2015446.

Nurmi et al. Lignans in selected wines. *Food Chem* **2003**, *83*, 303-9. doi:10.1016/S0308-8146(03)00222-X

Penalvo et al. Quantification of Lignans in Food Using Isotope Dilution Gas Chromatography/Mass Spectrometry. *J Agric Food Chem* **2008**, *53*, 9342-9347 doi: 10.1021/jf051488w

Smeds et al. Quantification of a broad spectrum of lignans in cereals, oilseeds, and nuts. *J Agric Food Chem* **2007**, *55*, 1337-46. doi: 10.1021/jf0629134

Tetens et al. Dietary intake and main sources of plant lignans in five European countries. *Food Nutr Res* **2013**, *57*, 19805 -doi: 10.3402/fnr.v57i0.19805

Thompson et al. Phytoestrogen Content of Foods Consumed in Canada, Including Isoflavones, Lignans, and Coumestan. *Nutr Cancer* **2006**, *54(2)*, 184-201, doi: 10.1207/s15327914nc5402_5

Valsta et al. Phyto-oestrogen database of foods and average intake in Finland. *Br J Nutr* **2003**, *89(S1)*, S31-S38. doi: 10.1079/BJN2002794.

Table S2. Dietary scales used to calculate dietary scores for DAI, GEN and ENL.

Table S2A, Scales for DAI and GEN associated to Urine determination;

Column /lines	B	C	D	E	F	G	H	I	J
1				**Dietary scores for DAI in urine**					
2		Two days before morning	Two days before noon	Two days before evening	Previous day morning	Previous noon	Previous evening	morning	Score
3	Soy juice	0.3	0.5	1.0	1.5	2.0	3.0	0.0	**=Σ(N3*N4)**
4			**0**	**0**	**0**	**0**	**0**	**0**	
5	Tofu /	0.3	0.5	1.0	1.5	2.0	3.0	0.0	**=Σ(N5*N6)**
6	soy-based cheese		**0**	**0**	**0**	**0**	**0**	**0**	
7	Soy-food	0.3	0.5	1.0	1.5	2.0	3.0	0.0	**=Σ(N7*N8)**
8			**0**	**0**	**0**	**0**	**0**	**0**	
9	Soy-based yogurt	0.0	0.1	0.3	0.5	1.0	1.5	0.0	**=Σ(N9*N10)**
10			**0**	**0**	**0**	**0**	**0**	**0**	
11	Transformed ++	0.0	0.1	0.2	0.4	0.8	1.5	0.0	**=Σ(N11*N12)**
12			**0**	**0**	**0**	**0**	**0**	**0**	
13	Transformed +	0.0	0.0	0.1	0.2	0.5	1.0	0.0	**=Σ(N13*N14)**
14			**0**	**0**	**0**	**0**	**0**	**0**	
15	Transformed	0.0	0.0	0.0	0.1	0.3	0.5	0.0	**=Σ(N15*N16)**
16			**1**	**0**	**0**	**1**	**2**	**0**	
17	Transformed -	0.0	0.0	0.0	0.0	0.0	0.0	0.0	**=Σ(N17*N18)**
19			**0**	**0**	**0**	**0**	**0**	**0**	
20	Canteen		0,1	0,1		0,2	0,2		**=Σ(N19*N20)**
21			**0**	**0**		**0**	**0**		
22							**Urine DAI Score**		**=Σ(J3;J20)**
23				**Dietary scores for GEN in urine**					
24		Two days before morning	Two days before noon	Two days before evening	Previous day morning	Previous noon	Previous evening	morning	Score
25	Soy juice	0.3	0.5	1.0	1.5	2.0	3.0	0.0	**=Σ(N25*N26)**
26			**0**	**0**	**0**	**0**	**0**	**0**	
27	Tofu /	0.3	0.5	1.0	1.5	2.0	3.0	0.0	**=Σ(N27*N28)**
25	soy-based cheese		**0**	**0**	**0**	**0**	**0**	**0**	
29	Soy-food	0.3	0.5	1.0	1.5	2.0	3.0	0.0	**=Σ(N29*N30)**
30			**0**	**0**	**0**	**0**	**0**	**0**	
31	Soy-based yogurt	0.0	0.1	0.3	0.5	1.0	1.5	0.0	**=Σ(N31*N32)**
32			**0**	**0**	**0**	**0**	**0**	**0**	
33	Transformed ++	0.0	0.1	0.2	0.4	0.8	1.5	0.0	**=Σ(N33*N34)**
34			**0**	**0**	**0**	**0**	**0**	**0**	
35	Transformed +	0.0	0.0	0.1	0.2	0.5	0.1	0.0	**=Σ(N35*N36)**
36			**0**	**0**	**0**	**0**	**0**	**0**	
37	Transformed	0.0	0.0	0.0	0.1	0.3	0.5	0.0	**=Σ(N37*N38)**
38			**0**	**0**	**0**	**0**	**0**	**0**	
39	Transformed -	0.0	0.0	0.0	0.0	0.0	0.0	0.0	**=Σ(N39*N40)**
40			**0**	**0**	**0**	**0**	**0**	**0**	
41	Canteen		0.1	0.1		0.2	0.2		**=Σ(N41*N42)**
42			**0**	**0**		**0**	**0**		

Urine GEN Score **=Σ(J25;J41)**

Urine IFs Score **=Σ(J22;J43)**

Table S2. Dietary scales used to calculate dietary scores for DAI, GEN and ENL.

Table S2B, Scales for DAI and GEN associated with plasma determination,

Column /lines	B	C	D	E	F	G	H	I	J
1				**Dietary score for DAI in plasma**					
2		Two days before morning	Two days before noon	Two days before evening	Previous day morning	Previous noon	Previous evening	morning	Score
3	Soy juice	0.5	1	2	3	4	8	8	=Σ(N3*N4)
4			0	0	0	0	0	0	
5	Tofu /	0.5	1	2	3	4	8	8	=Σ(N5*N6)
6	soy-based cheese		0	0	0	0	0	0	
7	Soy-food	0.5	1	2	3	4	8	8	=Σ(N7*N8)
8			0	0	0	0	0	0	
9	Soy-based yogurt	0	0.5	1	1.5	2	4	4	=Σ(N9*N10)
10			0	0	0	0	0	0	
11	Transformed ++	0	0.5	1	1.5	2	4	4	=Σ(N11*N12)
12			0	0	0	0	0	0	
13	Transformed +	0	0	0.5	0.75	1	2	2	=Σ(N13*N14)
14			0	0	0	0	0	0	
15	Transformed	0	0	0.25	0.4	0.5	1	1	=Σ(N15*N16)
16			0	0	0	0	0	0	
17	Transformed -	0	0	0	0	0	0	0	=Σ(N17*N18)
19			0	0	0	0	0	0	
20	Canteen		0.1	0.1		0.2	0.2		=Σ(N19*N20)
21			0	0	0	0	0	0	
22								**Plasma DAI Score**	**=Σ(J3:J20)/2**

Column /lines	B	C	D	E	F	G	H	I	J
23				**Dietary score for GEN in plasma**					
24		Two days before morning	Two days before noon	Two days before evening	Previous day morning	Previous noon	Previous evening	morning	Score
25	Soy juice	1	1.5	2	4	6	8	8	=Σ(N25*N26)
26			0	0	0	0	0	0	
27	Tofu /	1	1.5	2	4	6	8	8	=Σ(N27*N28)
25	soy-based cheese		0	0	0	0	0	0	
29	Soy-food	1	1.5	2	4	6	8	8	=Σ(N29*N30)
30			0	0	0	0	0	0	
31	Soy-based yogurt	0	0,5	1	2	3	4	4	=Σ(N31*N32)
32			0	0	0	0	0	0	
33	Transformed ++	0	0	0.5	1	1.5	2	2	=Σ(N33*N34)
34			0	0	0	0	0	0	
35	Transformed +	0	0	0.25	0.5	0.75	1	1	=Σ(N35*N36)
36			0	0	0	0	0	0	
37	Transformed	0	0.2	0.2		0.4	0.4	0	=Σ(N37*N38)
38			0	0	0	0	0	0	
39	Transformed -		0	0		0	0	0	=Σ(N39*N40)
40			0	0	0	0	0	0	
41	Canteen		0.1	0.1		0.2	0.2		=Σ(N41*N42)
42			0	0	0	0	0	0	
43								**Plasma GEN Score**	**=Σ(J25:J41)/2**
44									
45								**Plasma IFs Score**	**=Σ(J22;J43)/2**

Table S2. Dietary scales used to calculate dietary scores for DAI, GEN and ENL.

Table S2C, Scales for ENL associated with urine determination.

Column /lines	B	C	D	E	F	G	H	I	J
1		**Dietary Score for ENL in urine**							
2	Foodstuff	Two days before morning	Two days before noon	Two days before evening	Previous day morning	Previous day noon	Previous day evening	morning	Score
3	Cereals and grain	2	2	3	3	2	1	0	**=Σ(N3*N4)**
4	products	**0**	**0**	**0**	**0**	**0**	**0**	**0**	
5	Cabbages	2	2	3	3	2	1	0	**=Σ(N5*N6)**
6	Squashes	**0**	**0**	**0**	**0**	**0**	**0**	**0**	
7	Prunus / Apricots	2	2	3	3	2	1	0	**=Σ(N7*N8)**
8	Citrus / Dates	**0**	**0**	**0**	**0**	**1**	**0**	**0**	
9	Berries	2	2	3	3	2	1	0	**=Σ(N9*N10)**
10		**0**	**0**	**0**	**0**	**0**	**0**	**0**	
11	Chamomile	2	2	3	3	2	1	0	**=Σ(N11*N12)**
12		**0**	**0**	**0**	**0**	**0**	**0**	**0**	
13	Nuts	1	1	2	2	1	0.5	0	**=Σ(N13*N14)**
14	Oil seeds	**0**	**0**	**0**	**0**	**0**	**0**	**0**	
15	Beans / Lentils	1	1	2	2	1	0.5	0	**=Σ(N15*N16)**
16	Soy	**0**	**0**	**0**	**0**	**0**	**0**	**0**	
17	Fruits & vegetables	1	1	2	2	1	0.5	0	**=Σ(N17*N18)**
18	Pasta	**0**	**1**	**0**	**0**	**0**	**0**	**0**	
19	Coffee instant	1	1	2	2	1	0.5	0	**=Σ(N19*N20)**
20	Chocolate	**0**	**1**	**0**	**0**	**0**	**0**	**0**	
21	Carob /	1	1	2	2	1	0.5	0	**=Σ(N21*N22)**
22	Transformed meals	**0**	**0**	**0**	**0**	**0**	**0**	**0**	
23	Wine / Beer	0.5	1	1	1	0.5	0.2	0	**=Σ(N23*N24)**
24		**0**	**0**	**0**	**0**	**0**	**0**	**0**	
25	Cantine	0.2	0.5	1	1	0.5	0.2	0	**=Σ(N25*N26)**
26		**0**	**0**	**0**		**0**	**0**		
27								**Urine score Enterolactone**	**=Σ(J3:J25)**

Table S2D, Scales for ENL associated with plasma determination.

Column /lines	B	C	D	E	F	G	H	I	J
1		**Dietary Score for ENL in plasma**							
2	Foodstuff	Two days before morning	Two days before noon	Two days before evening	Previous day morning	Previous day noon	Previous day evening	morning	Score
3	Cereals and grain	0.2	0.2	1	3	2	1	0	**=Σ(N3*N4)**
4	products		**0**	**0**	**0**	**0**	**0**	**0**	
5	Cabbages	0.2	0.5	1	3	2	1	0	**=Σ(N5*N6)**
6	Squashes		**0**	**0**	**0**	**0**	**0**	**0**	
7	Prunus / Apricots	0.2	0.5	1	3	2	1	0	**=Σ(N7*N8)**
8	Citrus / Dates		**0**	**0**	**0**	**1**	**0**	**0**	
9	Berries	0.2	0.5	1	3	2	1	0	**=Σ(N9*N10)**
10			**0**	**0**	**0**	**0**	**0**	**0**	
11	Chamomile	0.2	0.5	1	3	2	1	0	**=Σ(N11*N12)**
12			**0**	**0**	**0**	**0**	**0**	**0**	
13	Nuts	0	0.2	0.5	2	1	0.5	0	**=Σ(N13*N14)**
14	Oil seeds		**0**	**0**	**0**	**0**	**0**	**0**	
15	Beans / Lentils	0	0.2	0.5	2	1	0.5	0	**=Σ(N15*N16)**
16	Soy		**0**	**0**	**0**	**0**	**0**	**0**	
17	Fruits & vegetables	0	0.2	0.5	2	1	0.5	0	**=Σ(N17*N18)**
18	Pasta		**1**	**0**	**0**	**0**	**0**	**0**	
19	Coffee instant	0	0.2	0.5	2	1	0.5	0	**=Σ(N19*N20)**
20	Chocolate		**1**	**0**	**0**	**0**	**0**	**0**	
21	Carob /	0	0.2	0.5	2	1	0.5	0	**=Σ(N21*N22)**
22	Transformed meals		**0**	**0**	**0**	**0**	**0**	**0**	
23	Wine / Beer	0	0	0.2	1	0.5	0.2	0	**=Σ(N23*N24)**
24			**0**	**0**	**0**	**0**	**0**	**0**	
25	Cantine	0	0	0.2	1	0.5	0.2	0	**=Σ(N25*N26)**
26			**0**	**0**		**0**	**0**		
27								**Plasma score Enterolactone**	**=Σ(J3:J25)**

Figure S1. Consort diagram of the recruitment of volunteers at the time of the study samples collected and inquiry completed.

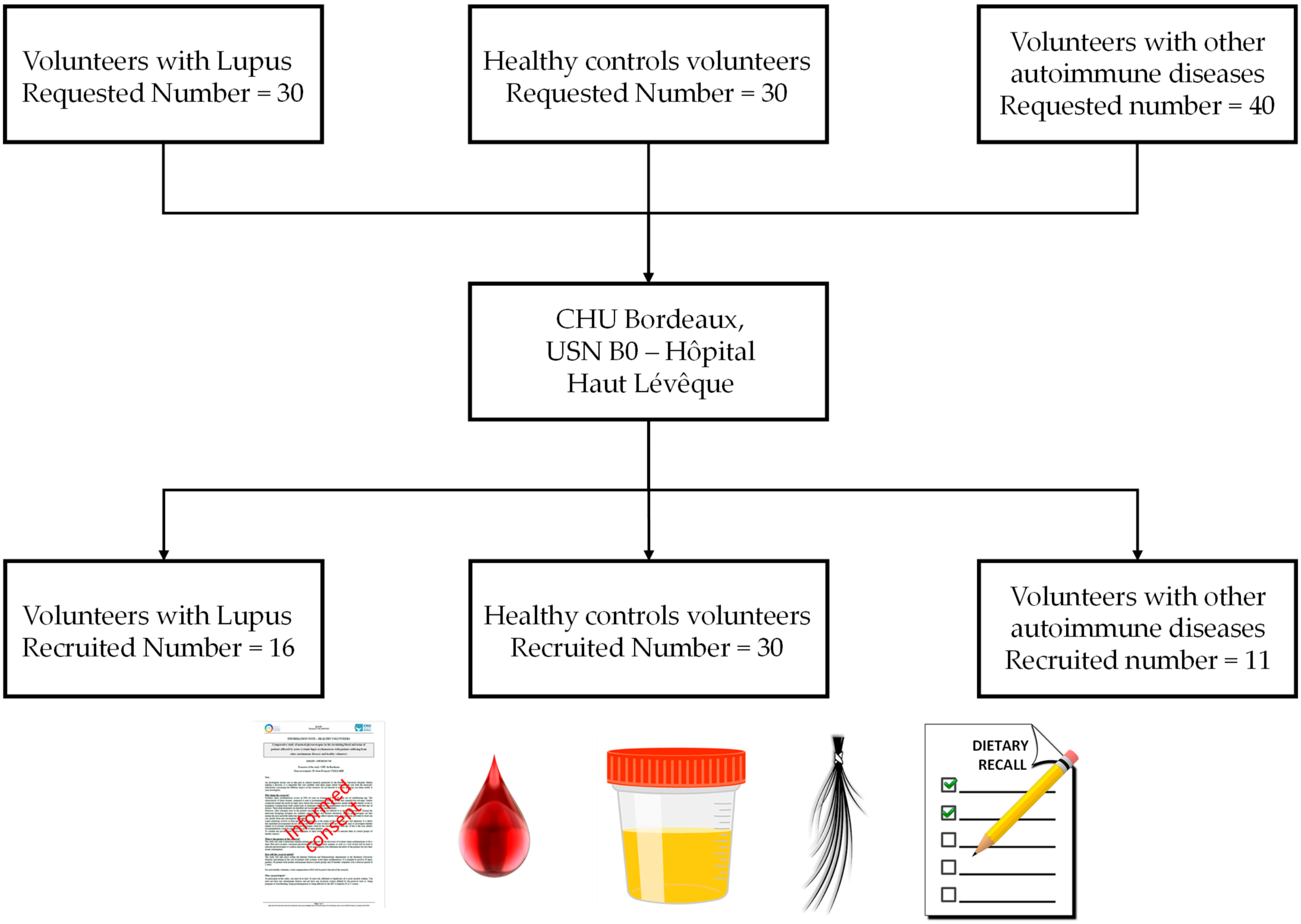

Figure S2. Correlation between urinary DAI and GEN and their respective dietary scores. **Fig S2A**: correlation between urinary DAI concentrations and the corresponding dietary scores. **Fig S2B**: correlation between urinary GEN concentrations and the corresponding dietary scores.

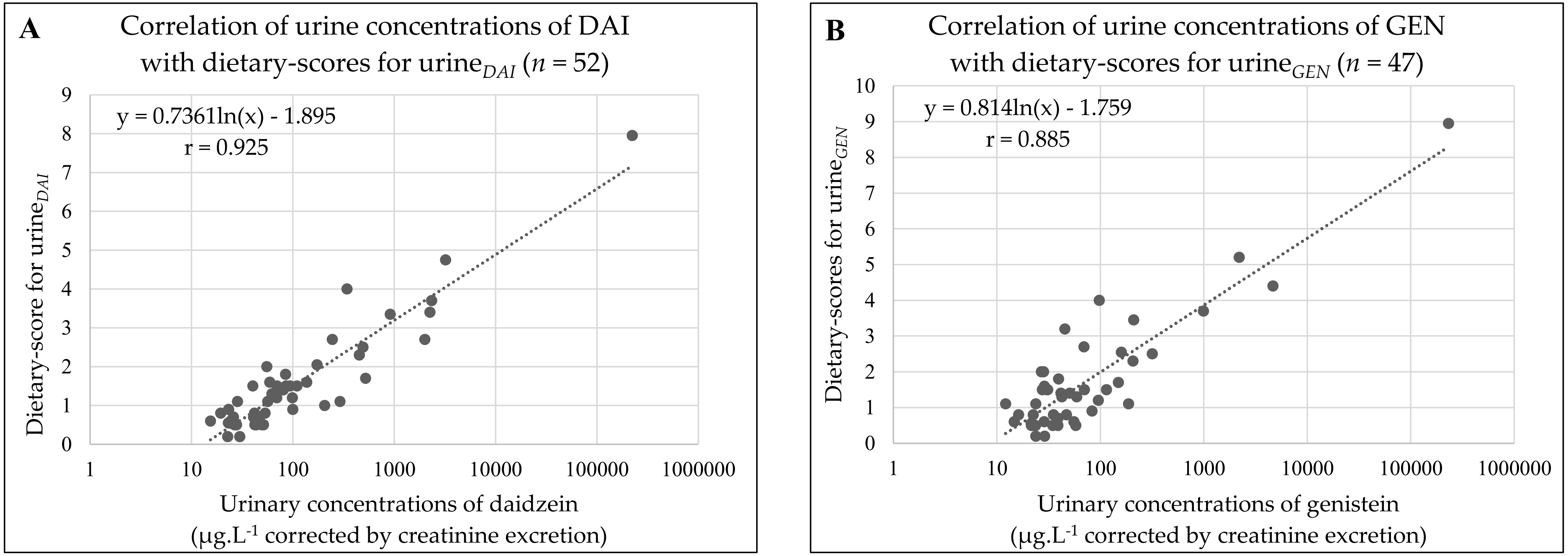

Figure S3. Correlation between plasma DAI and GEN and their respective dietary scores. **Fig S3A**: correlation between plasma DAI concentrations and the corresponding dietary scores. **Fig S3B**: correlation between plasma GEN concentrations and the corresponding dietary scores.

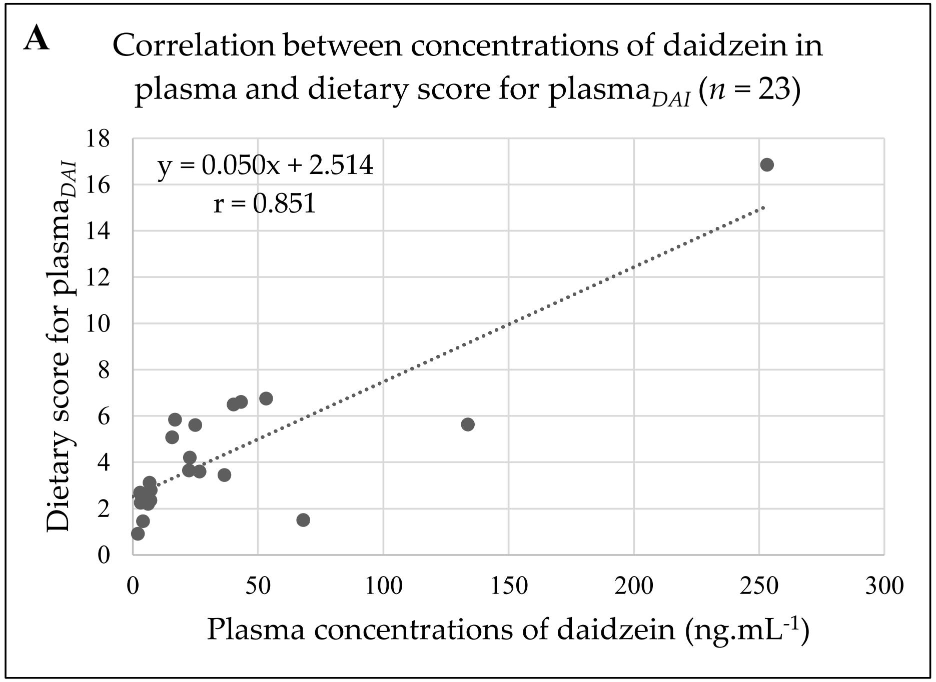

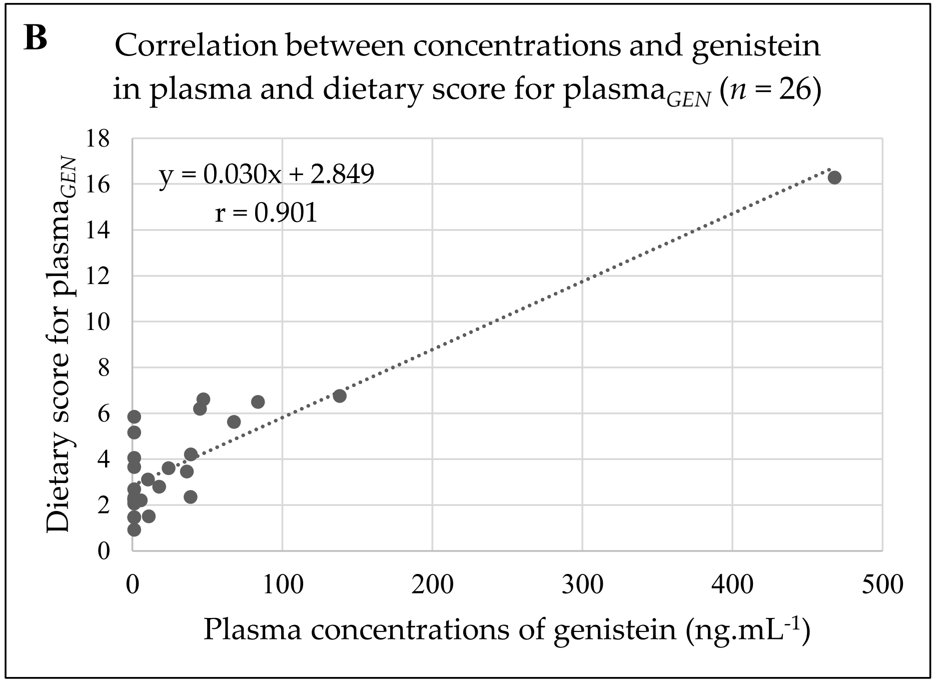

Figure S4. Correlation between urine and plasma IFs concentrations.

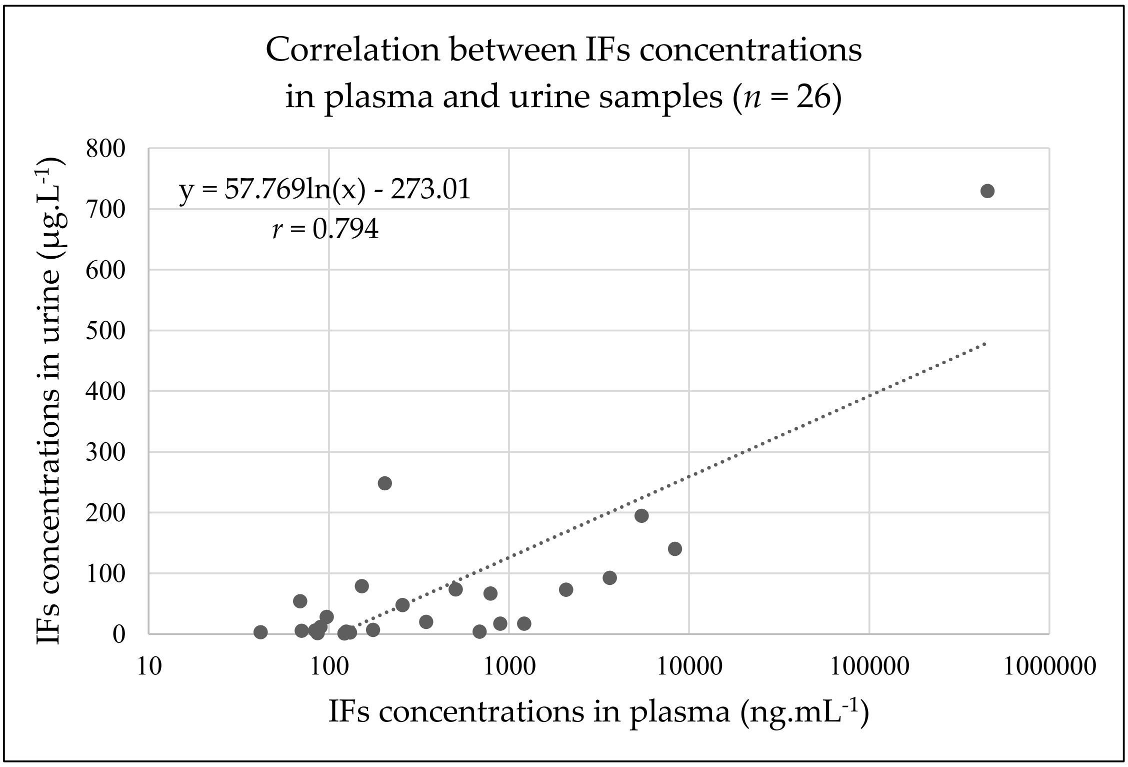

Figure S5. Correlation between ENL and IFs data. **Fig S5A**. correlation based on PHYTOs urine concentrations; **Fig S5B**. correlation based on PHYTOs dietary scores.

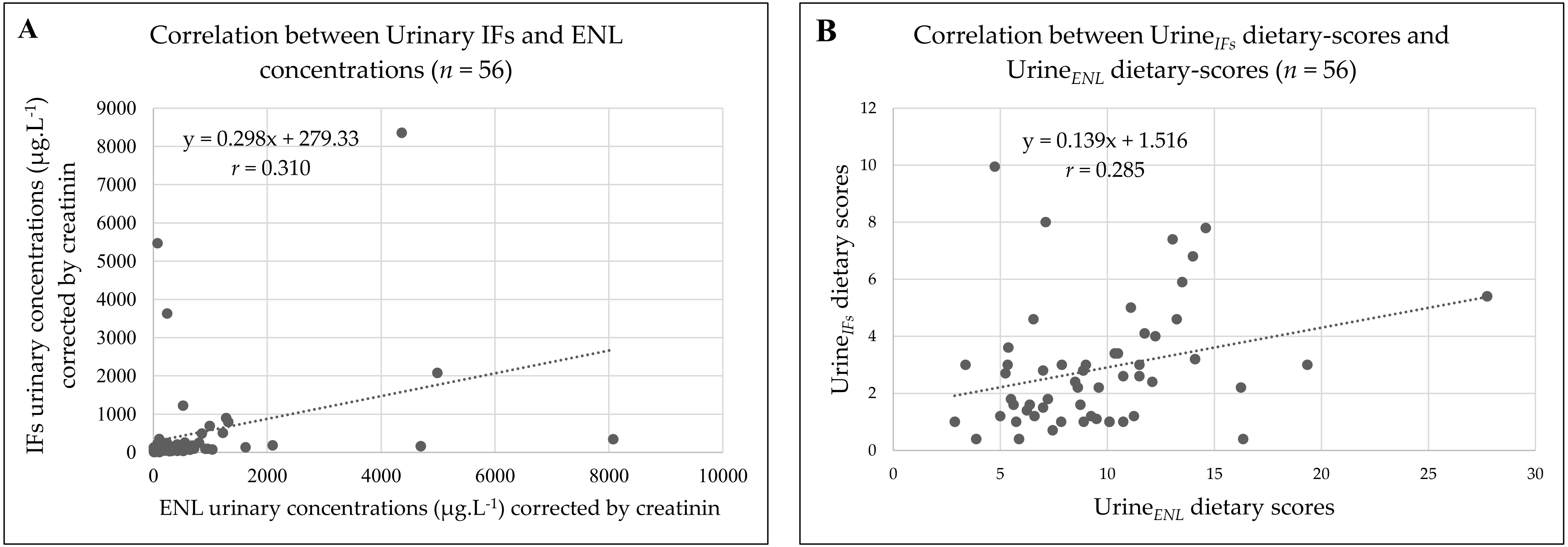

Document D1. Open questionnaire proposed for the last 24 hours recall

Diet inquiry: Recall of the previous day (this morning)

Subject identification

Study acronyme		Promotor name	
Subject code		Date of the inquiry	
Investigator name		Name of the inquirer	

Breakfast consumption

Meal time

Meal location — At home ☐ — Outside Precise

		Nbre	Home made	Trade mark	Total consumption	
Hot drink(s)		☐	☐		☐ 1/2	☐ Spoon
		☐	☐		☐ 1	☐ Glass
		☐	☐		☐ 2	☐ Bowl
					☐ 3	☐ Mug
					☐ 4	☐ Portion
Cold drink(s)		☐	☐		☐ 1/2	☐ Spoon
		☐	☐		☐ 1	☐ Glass
		☐	☐		☐ 2	☐ Bowl
					☐ 3	☐ Mug
					☐ 4	☐ Portion
Cereals		☐	☐		☐ 1/2	☐ Spoon
		☐	☐		☐ 1	☐ Plate
		☐	☐		☐ 2	☐ Bowl
					☐ 3	☐ Mug
					☐ 4	☐ Portion
Bread products (sandwich bread, brioche, pastry …)		☐	☐		☐ 1/2	☐ Spoon
		☐	☐		☐ 1	☐ Slice
		☐	☐		☐ 2	☐
					☐ 3	☐ Plate
					☐ 4	☐ Portion
Delicatessen		☐	☐		☐ 1/2	☐ Spoon
		☐	☐		☐ 1	☐ Slice
		☐	☐		☐ 2	☐ Toast
					☐ 3	☐ Plate
					☐ 4	☐ Portion

Meal time ☐

		Nbre	Home made	Trade mark	Total consumption	
Eggs and derived products		☐	☐		☐ 1/2	☐ Spoon
		☐	☐		☐ 1	☐ Glass
		☐	☐		☐ 2	☐ Bowl
					☐ 3	☐ Plate
					☐ 4	☐ Portion

Diet inquiry: Recall of the previous day (this morning)

Subject identification

**Study acronyme**		**Promotor name**	
**Subject code**		**Date of the inquiry**	
**Investigator name**		**Name of the inquirer**	

Breackfast consumption (following)

**Dairy products (animal or plant-based milk)**						1/2<br>1<br>2<br>3<br>4		Spoon<br>Glass<br>Bowl<br>Plate<br>Portion
**Fruit products**						1/2<br>1<br>2<br>3<br>4		Spoon<br>Glass<br>Bowl<br>Plate<br>Portion
**Vegetables (Beans, potatoes)**						1/2<br>1<br>2<br>3<br>4		Spoon<br>Glass<br>Bowl<br>Plate<br>Portion
**Other (sweets, biscuits, chocolate …)**						1/2<br>1<br>2<br>3<br>4		Spoon<br>Glass<br>Bowl<br>Plate<br>Portion
**Trimmings (Butter, cream, Jam...)**						1/2<br>1<br>2<br>3<br>4		Spoon<br>Glass<br>Bowl<br>Plate<br>Portion

Diet inquiry: Recall of the previous day (yesterday)

Subject identification

Study acronyme	☐	Promotor name	
Subject code	☐	Date of the inquiry	
Investigator name	☐	Name of the inquirer	

Dinner consumption

Meal time

Meal location — At home ☐ — Outside Precise

		Home made	Trade mark	Total consumption	
Starter		☐		☐ 1/2 ☐ 1 ☐ 2 ☐ 3 ☐ 4	☐ Spoon ☐ Glass ☐ Bowl ☐ Plate ☐ Portion
Meal 1		☐		☐ 1/2 ☐ 1 ☐ 2 ☐ 3 ☐ 4	☐ Spoon ☐ Glass ☐ Bowl ☐ Plate ☐ Portion
Meal 2		☐		☐ 1/2 ☐ 1 ☐ 2 ☐ 3 ☐ 4	☐ Spoon ☐ Glass ☐ Bowl ☐ Plate ☐ Portion
Meal 3		☐		☐ 1/2 ☐ 1 ☐ 2 ☐ 3 ☐ 4	☐ Spoon ☐ Glass ☐ Bowl ☐ Plate ☐ Portion
Cheese		☐		☐ 1/2 ☐ 1 ☐ 2 ☐ 3 ☐ 4	☐ Spoon ☐ Glass ☐ Bowl ☐ Plate ☐ Portion
Dessert		☐		☐ 1/2 ☐ 1 ☐ 2 ☐ 3 ☐ 4	☐ Spoon ☐ Glass ☐ Bowl ☐ Plate ☐ Portion
Trimmings Sauce, bread,		☐		☐ 1/2 ☐ 1 ☐ 2 ☐ 3	☐ Spoon ☐ Glass ☐ Bowl ☐ Plate

Diet inquiry: Recall of the previous day (yesterday)

Subject identification

**Study acronyme**	☐	**Promotor name**	
**Subject code**	☐	**Date of the inquiry**	
**Investigator name**	☐	**Name of the inquirer**	

Dinner consumption **Meal time**

**Alcoholic beverages**		☐		☐ 1/2<br>☐ 1<br>☐ 2<br>☐ 3<br>☐ 4	☐ Spoon<br>☐ Glass<br>☐ Bowl<br>☐ Plate<br>☐ Portion
**Non-alcoholic bevrages (juice, soda, milk coffee, tea…)**		☐		☐ 1/2<br>☐ 1<br>☐ 2<br>☐ 3<br>☐ 4	☐ Spoon<br>☐ Glass<br>☐ Bowl<br>☐ Bottle
**Water**		☐		☐ 1/2<br>☐ 1<br>☐ 2<br>☐ 3<br>☐ 4	☐ Spoon<br>☐ Glass<br>☐ Bowl<br>☐ Bottle
**Others (sweets, biscuits chocolate …)**		☐		☐ 1/2<br>☐ 1<br>☐ 2<br>☐ 3<br>☐ 4	☐ Spoon<br>☐ Unit<br>☐ Handle<br>☐ Plate<br>☐ Portion

Diet inquiry: Recall of the previous day (yesterday)

Subject identification

Study acronyme	☐	Promotor name	
Subject code	☐	Date of the inquiry	
Investigator name	☐	Name of the inquirer	

Lunch consumption

Meal time

Meal location — At home ☐ — Outside Precise

		Home made	Trade mark	Total consumption	
Starter		☐ ☐ ☐		☐ 1/2 ☐ 1 ☐ 2 ☐ 3 ☐ 4	☐ Spoon ☐ Glass ☐ Bowl ☐ Plate ☐ Portion
Meal 1		☐		☐ 1/2 ☐ 1 ☐ 2 ☐ 3 ☐ 4	☐ Spoon ☐ Glass ☐ Bowl ☐ Plate ☐ Portion
Meal 2		☐		☐ 1/2 ☐ 1 ☐ 2 ☐ 3 ☐ 4	☐ Spoon ☐ Glass ☐ Bowl ☐ Plate ☐ Portion
Meal 3		☐		☐ 1/2 ☐ 1 ☐ 2 ☐ 3 ☐ 4	☐ Spoon ☐ Glass ☐ Bowl ☐ Plate ☐ Portion
Cheese		☐		☐ 1/2 ☐ 1 ☐ 2 ☐ 3 ☐ 4	☐ Spoon ☐ Glass ☐ Bowl ☐ Plate ☐ Portion
Dessert		☐		☐ 1/2 ☐ 1 ☐ 2 ☐ 3 ☐ 4	☐ Spoon ☐ Glass ☐ Bowl ☐ Plate ☐ Portion
Trimmings Sauce, bread,		☐		☐ 1/2 ☐ 1 ☐ 2 ☐ 3	☐ Spoon ☐ Toast ☐ Bowl ☐ Plate

Diet inquiry: Recall of the previous day (yesterday)

Subject identification

**Study acronyme**		**Promotor name**	
**Subject code**		**Date of the inquiry**	
**Investigator name**		**Name of the inquirer**	

Lunch consumption (following) **Meal time**

**Alcoholic beverages**				1/2<br>1<br>2<br>3<br>4	Glass<br>Mug<br>Bowl<br>Bottle
**Non-alcoholic bevrages (juice, soda, milk coffee, tea…)**				1/2<br>1<br>2<br>3<br>4	Glass<br>Mug<br>Bowl<br>Bottle
**Water**				1/2<br>1<br>2<br>3<br>4	Glass<br>Mug<br>Bowl<br>Bottle
**Others (sweets, biscuits chocolate …)**				1/2<br>1<br>2<br>3<br>4	Spoon<br>Unit<br>Handle<br>Plate<br>Portion

Diet inquiry: Recall of the previous day (yesterday)

Subject identification

Study acronyme

Subject code

Investigator name

Promotor name

Date of the inquiry

Name of the inquirer

Out of meal intakes

Intake time

Intake place

At home

Outside

Precise

Home made

Trade mark

Portion size

Product name

1/2
1
2
3
4

Spoon
Glass
Bowl
Plate
Portion

Intake time

Intake place

At home

Outside

Precise

Home made

Trade mark

Portion size

Product name

1/2
1
2
3
4

Spoon
Glass
Bowl
Plate
Portion

Intake time

Intake place

At home

Outside

Precise

Home made

Trade mark

Portion size

Product name

1/2
1
2
3
4

Spoon
Glass
Bowl
Plate
Portion

Intake time

Intake place

At home

Outside

Precise

Home made

Trade mark

Portion size

Product name

1/2
1
2
3
4

Spoon
Glass
Bowl
Plate
Portion

Document D2. Example of an inform consent form (Translated in English)

INFORMATION NOTE – HEALTHY VOLUNTEERS

Comparative study of natural phytoestrogens in the circulating blood and urine of patients affected by acute systemic lupus erythematosus with patients suffering from other autoimmune diseases and healthy volunteers

ISOLED – CHUBX2017/08

Promotor of the study: **CHU de Bordeaux**
Main investigator: **Pr Jean-François VIALLARD**

Dear,

An investigator invites you to take part in clinical research sponsored by the Bordeaux University Hospital. Before making a decision, it is important that you carefully read these pages which will provide you with the necessary information concerning the different aspects of this research. Do not hesitate to ask any questions you deem useful to your investigator.

Why doing this research?
Systemic lupus erythematosus occurs in 90% of cases in women and 90% of them are of childbearing age. The characteristic of these women, compared to men or postmenopausal women, is that they manufacture estrogen. Studies conducted around the world on lupus have shown that estrogen, whether endogenous (made during the female cycle) or exogenous (coming from birth control pills or hormonal treatments for menopause) can be associated with flare-ups of disease. These drug treatments are therefore not recommended for lupus patients.
However, other estrogens exist in the patient's environment which are referred to as endocrine disruptors. Among the endocrine disrupting estrogens are synthetic substances but also natural substances. Dietary phytoestrogens are thus among the most powerful endocrine disruptors with estrogenic effects known today. The quantities provided by food can vary greatly from one consumption to another.
Lupus pathology evolves in flare-ups and the phenomena at the origin of these flare-ups are still unknown. It is likely that unstudied environmental factors may be the cause of some of these flare-ups. The study aims to investigate whether regular or occasional consumption of phytoestrogens could be the cause of disease flare-ups. If this is the case, dietary recommendations could be systematically given to lupus patients.
To validate the possible role of phytoestrogens in lupus disease, we also need to measure them in control groups of healthy subjects.

What is the purpose of this research?
The study will seek to determine whether patients who consult for the discovery of systemic lupus erythematosus or for a lupus flare have recently consumed phytoestrogens. Blood and urine samples, as well as a lock of hair will be used to measure phytoestrogens to confirm exposure. A food questionnaire will determine the habits of the patients but also their recent consumption.

How will this research unfold?
The study will take place within the Internal Medicine and Rheumatology departments at the Bordeaux University Hospital specializing in the care of patients with systemic acute lupus erythematosus. It is planned to involve 30 lupus patients, 40 patients with another autoimmune disease (control group) and 30 healthy volunteers over a forecast period of 2 years.

For each healthy volunteer, a total compensation of €30 will be paid at the end of the research.

Who can participate?
To participate in this study, you must be at least 18 years old, affiliated or beneficiary of a social security scheme. You must not have any autoimmune disease, and not have any exclusion criteria defined by the protocol such as: being pregnant or breastfeeding, being postmenopausal or being affected by the HIV or hepatitis B or C viruses..

Cette note d'information/formulaire de consentement a été conçue et rédigée à partir du document-type du CHU de Bordeaux version 4.0 du 31/03/2017 basé sur le modèle du GIRCI SOHO

Thus, as part of this study, HIV, hepatitis B or C serologies will be carried out, as well as a search for anti-nucleus antibodies. If one of these results is positive, you will be immediately informed of the result and a specialist consultation will then be advised.

What will you be asked?

After reading this information note and talking with your doctor, you will have a reflection period after which you will have to sign your consent form if you decide to take part in this study.

After signing the consent form, a blood test will be carried out and 4 additional tubes of blood, a urine sample and a strand of hair for research will be taken. You will also be asked to complete an eating habits questionnaire and a 48-hour reminder (about 20 minutes).

The table below describes the visit process:.

	Pre-inclusion (J-2 to M-1)	Inclusion T 0[£]
Information on the research project	✓	
Check of the inclusion criteria	✓	✓
Collection of informed consent		✓
Clinical examination		✓
Biology checking[1]		✓
bHCG measurements		✓
Dietary inquiry (average 20 min)		✓
Biological sampling[2]		✓

£ If possible 1 week after start of menstrual bleeding
1 Biology checking : HIV serology, B and C hepatitis nuclear antibody analysis.
2 4 blood tubes (i.e. 25ml) and a urine sample (10 ml) and a hair sample (a pen diameter).

The study includes a biological collection
The samples taken will constitute a bank of serum, plasma, DNA, urine and hair kept as part of this research, to meet the objective of the latter under the responsibility of Pr Jean-François Viallard, principal investigator of the research, and responsible for the collection. This collection will be produced in accordance with the regulations and declared to the competent authorities.
The collection will be kept in the Biological Resource Center of the Bordeaux University Hospital located in Tondu, Place Amélie Raba-Léon 33076 Bordeaux Cedex. The data corresponding to the samples taken will be anonymized.
At the end of the research, the collection of biological samples constituted can be used for possible future studies.
You are free to refuse to participate in this collection and you can therefore request the destruction of your blood samples at any time.
You will be able to be informed of the results obtained and of the fate of your samples, including in the event that you no longer wish to take part in this research.

Role of genetic determinants
Some research on your biological samples may focus on the role of genetic determinants; which will require an examination of your genetic characteristics without an identifying purpose. For this research your written consent is required. You can express this by signing a specific consent which will be given to you by your doctor.

What are the expected benefits?
If the hypothesis tested in this study turns out to be correct, that is to say that the intake of phytoestrogens is linked to the declaration of LEAD or to the appearance of lupus flare-ups, dietary advice will be given to patients. lupus and help in identifying risk-inducing products will be provided. This information will help reduce the incidence of relapses.
The research in which you will participate therefore has a vocation of secondary prevention. Ultimately, the demonstration of the aggravating effects of phyto-oestrogens on LEAD could lead to a review of the regulations on these compounds and encourage manufacturers to reduce the phyto-oestrogen content of food.

What are the possible disadvantages?
We will ask you to take four (4) additional tubes of blood (approximately 25ml), 10ml of urine and a lock of hair on inclusion in the study for the constitution of the biological collection necessary for this research.
This additional sample of approximately 25 ml of blood will have no impact on your state of health. There will be no trial of treatment and no invasive examination as part of this study.

What are your rights ?
The investigator who offers you to take part in this research must provide you with all the necessary explanations concerning this research. If you wish to withdraw from it at any time whatsoever, and for whatever reason, without having to justify yourself.

If during the research, you no longer wish to participate, the data / samples collected before the withdrawal of your consent may be kept and used in the context of the research, unless you object. In this case, they will be destroyed.

Confidentiality and processing of personal data
As part of the research, computer processing of your personal data will be implemented to allow analysis of the results of the research with regard to the objectives presented to you.
The data controller is Bordeaux University Hospital.
The study investigator and other authorized study personnel will collect information about you and after obtaining your consent. Only the information strictly necessary for the purpose of the research will be collected. This information will relate to your health and your participation in the study. They may also concern special categories of personal data such as genetic data, lifestyle and ethnic origin if the collection of this data is justified by the study. This information is reported on files specific to the study, called observation books, provided by the Promoter.
The collection of this data will be done for the purposes of scientific research and their processing will have the purpose of meeting the objectives of this research.
These data will be kept for the duration of the study until the final report or until the last publication and then archived for a period in accordance with the regulations..

In order to ensure the confidentiality of your personal information, neither your name nor any other information which would make it possible to identify you directly will be entered in the observation notebook or any other file or sample that the investigator of the study will provide. to the Promoter or authorized representatives of the Promoter. You will only be identified by a code and your initials. The code is used so that the study investigator can identify you if necessary. This data will be stored on secure computer servers.

In accordance with the provisions of the law relating to data processing, files and freedoms (law n ° 78-17 of January 6, 1978 relating to data processing, files and freedoms modified by law n ° 2018-493 of 20 June 2018 relating to the protection of personal data) and the General Data Protection Regulation (EU Regulation 2016/679), you have the right to access and rectify your personal information. In some cases, you can also request the restriction of the processing of your personal information, object to certain types of processing, request that your personal information be erased and request that your personal information be provided to you, or provided to a third party, in a digital format (right of portability). In accordance with the law, certain data collected may not be deleted if this deletion makes it impossible or seriously compromises the achievement of the research objectives. You can exercise these rights by requesting it in writing from the study investigator who will forward the request to the sponsor. The promoter will respond to your requests in accordance with its legal and regulatory obligations.

The Promoter may disclose personal information to regulatory agencies or its research partners. These people, companies and agencies may be located in France, other European Economic Area (EEA) countries, the United States and other countries outside the EEA. It is possible that some countries outside the EEA do not offer the same level of privacy protection as in France. In this case, the Promoter will however maintain the confidentiality of all personal information that it exchanges within the limits of the law. The Promoter will adopt appropriate contractual measures relating to the protection and transfer of data, to ensure that the relevant recipients outside the EEA provide an adequate level of protection regarding your personal information and in accordance with the law.
You have the right to object to the transmission of data covered by professional secrecy that may be used in the context of this research and to be processed. You can also access all of your medical data directly or through the doctor of your choice in accordance with the provisions of Article L1111-7 of the Public Health Code. These rights are exercised with the doctor who follows you in the context of the research and who knows your identity.

The competent authorities and the Sponsor or its authorized representatives may also need to access your medical records and your study file, in order to verify the quality of the data collected within the framework of the study.

Your coded personal information may be used for other scientific research on your disease or other diseases always in accordance with applicable laws and regulations.

For any questions about the processing of your personal data or your rights associated with this data, you can contact the data protection officer of the Bordeaux University Hospital (dpo@chu-bordeaux.fr).

If, despite the measures put in place by the Promoter, you believe that your rights have not been respected, you can file a complaint with the data protection supervisory authority, the Commission Nationale de l'Informatique et des Libertés (CNIL).

Regulatory Considerations:
In accordance with law n°2012-300 of March 5, 2012 relating to research involving the human person:
- this research obtained the favorable opinion of the Committee for the Protection of People Ile de France V and was the subject of information from the National Agency for the Safety of Medicines and Health Products (ANSM),
- the Promoter of this research, the Bordeaux University Hospital (12, rue Dubernat, 33404 Talence cedex), has taken out civil liability insurance with HDI Global SE (n°0100665414025-170026),
- the Promoter assumes compensation for the harmful consequences of the research involving the human person.

Search results :
When this research is complete, you will be personally informed of the overall results by your doctor as soon as these are available and if you wish.

After reading this information note, feel free to ask your investigator any questions you want. After a period of reflection, if you agree to participate in this research, you must complete and sign the consent to participate form. A copy of the complete document will be given to you.

Thank you for your attention to this information note..

INFORMED CONSENT FORM – HEALTHY VOLUNTEERS

Comparative study of natural phytoestrogens in the circulating blood and urine of patients affected by acute systemic lupus erythematosus with patients suffering from other autoimmune diseases and healthy volunteers

ISOLED – CHUBX2017/08

Promotor of the study: **CHU de Bordeaux**
Main investigator: **Pr Jean-François VIALLARD**

I, the undersigned) ....(surname, first name) certify that I have read and understood the information note that was given to me.

I had the opportunity to ask all the questions I wanted to Pr/Dr .... (surname, first name) who explained to me the nature, the objectives, the potential risks and the constraints linked to my participation in this research.

I am aware of the possibility reserved for me to interrupt my participation in this research at any time without having to justify my decision and I will do my best to inform the investigator who is following me in the research.

I had the assurance that the decisions necessary for my health will be taken at all times, in accordance with the current state of medical knowledge.

I am aware that this research has received the favorable opinion of a Committee for the Protection of Persons and has been the subject of information from the ANSM.

The Bordeaux University Hospital has signed a commitment to comply with the "Reference Methodology" (MR-001) pursuant to the provisions of Article 54 paragraph 5 of the amended law of January 6, 1978 relating to information, files and to freedoms.

The sponsor of the research (CHU de Bordeaux, 12 rue Dubernat 33404 Talence Cedex) has taken out civil liability insurance in the event of damage with the company HDI Global SE.

I agree that the people who collaborate in this research or who are mandated by the sponsor, as well as possibly the representative of the Health Authorities, have access to the information with the strictest respect for confidentiality.

I accept that the data recorded during this research may be subject to computerized processing under the responsibility of the promoter. I am informed that at the end of this research, these data may be used for other research purposes (new statistical analyses) and I have been informed of my right to oppose.

I am informed of the possibility that part of the samples taken during this research protocol will be kept for 5 years for subsequent use for research purposes and I have been informed of my right to oppose it.

I am informed that part of the samples taken during this research protocol will be kept in a biobank for 5 years for research purposes. I have been informed of my right to consent or not.

I have noted that, in accordance with the General Data Protection Regulations (GDPR) and the provisions of the law relating to data processing, files and freedoms, I have a right of access, rectification, deletion, limitation of processing, data portability, opposition and withdrawal. These rights are exercised with the investigator who follows me in the context of this research and who knows my identity.

My consent in no way relieves the investigator and the research sponsor of their responsibilities towards me. I retain all rights guaranteed by law.

The overall results of the research will be communicated to me directly, if I so wish, in accordance with the law of March 4, 2002 relating to the rights of patients and the quality of the health system.

Having had sufficient time to reflect before making my decision, I freely and voluntarily agree to participate in the ISOLED research

I freely and voluntarily agree that my data (including biological) may be stored and used for other research purposes (new statistical analyses) |__| **yes** |__| **no**

I may at any time request additional information from the investigator who offered me to participate in this research, telephone number:

Done in: ________________________ on:	Done in: ________________________ on:
Signature of the participant :	Signature of the investigator :

Cette note d'information/formulaire de consentement a été conçue et rédigée à partir du document-type du CHU de Bordeaux version 4.0 du 31/03/2017 basé sur le modèle du GIRCI SOHO
